# Harnessing infrared thermography and multi-convolutional neural networks for early breast cancer detection

**DOI:** 10.1038/s41598-025-09330-2

**Published:** 2025-07-28

**Authors:** Omneya Attallah

**Affiliations:** 1 Department of Electronics and Communications Engineering, College of Engineering and Technology, Arab Academy for Science, Technology and Maritime Transport, Alexandria, 21937 Egypt; 2 Wearables, Biosensing, and Biosignal Processing Laboratory, Arab Academy for Science, Technology and Maritime Transport, Alexandria, 21937 Egypt

**Keywords:** Computer aided diagnois (CAD), Deep learning, Breast cancer, Non-negative matrix factorization, Relief-F feature selection, Thermograms, Convolutional neural networks, Biomedical engineering, Computational science, Computer science

## Abstract

Breast cancer is a relatively common carcinoma among women worldwide and remains a considerable public health concern. Consequently, the prompt identification of cancer is crucial, as research indicates that 96% of cancers are treatable if diagnosed prior to metastasis. Despite being considered the gold standard for breast cancer evaluation, conventional mammography possesses inherent drawbacks, including accessibility issues, especially in rural regions, and discomfort associated with the procedure. Therefore, there has been a surge in interest in non-invasive, radiation-free alternative diagnostic techniques, such as thermal imaging (thermography). Thermography employs infrared thermal sensors to capture and assess temperature maps of human breasts for the identification of potential tumours based on areas of thermal irregularity. This study proposes an advanced computer-aided diagnosis (CAD) system called Thermo-CAD to assess early breast cancer detection using thermal imaging, aimed at assisting radiologists. The CAD system employs a variety of deep learning techniques, specifically incorporating multiple convolutional neural networks (CNNs) to enhance diagnostic accuracy and reliability. To effectively integrate multiple deep features and diminish the dimensionality of features derived from each CNN, feature transformation and selection methods, including non-negative matrix factorization and Relief-F, are used leading to a reduction in classification complexity. The Thermo-CAD system is assessed utilising two datasets: the DMR-IR (Database for Mastology Research Infrared Images), for distinguishing between normal and abnormal breast tissues, and a novel thermography dataset to distinguish abnormal instances as benign or malignant. Thermo-CAD has proven to be an outstanding CAD system for thermographic breast cancer detection, attaining 100% accuracy on the DMR-IR dataset (normal versus abnormal breast cancer) using CSVM and MGSVM classifiers, and lower accuracy using LSVM and QSVM classifiers. However, it showed a lower ability to distinguish benign from malignant cases (second dataset), achieving an accuracy of 79.3% using CSVM. Yet, it remains a promising tool for early-stage cancer detection, especially in resource-constrained environments.

## Introduction

Breast cancer is a common carcinoma, primarily affecting women worldwide^1^. The World Health Organisation projected that in 2020, about 2.3 million instances of breast cancer had been identified in women globally, leading to 685,000 deaths^2^. Breast self-exams and clinical evaluations are essential methods for detecting breast cancer. Alongside diagnostic procedures and physical assessments, medical imaging is crucial for the early detection of breast cancer. Various screening techniques, including magnetic resonance imaging (MRI), ultrasound, mammography, and histopathology are commonly employed for breast cancer detection. Mammography is commonly used as it is especially proficient in identifying microcalcifications, bilateral asymmetry, speculated masses, and morphological distortion by radiologists^3^. However, it is ineffective in cases of dense breast tissue^4^. Conversely, ultrasound imaging has a limited ability to differentiate between benign and malignant masses, often leading to false positives. MRI, although efficacious, necessitates costly apparatus^5^, and its confined setting may induce discomfort for numerous patients^6^. While biopsy, the examination of tissue samples under a microscope, is regarded as the gold standard for breast cancer diagnosis, it presents some challenges. This encompasses its invasive and time-intensive characteristics^7^, as well as the requirement for costly microscopes and highly skilled professionals^8^.

Thermography is a recent scanning technique that captures infrared radiation generated by human organs. This method can be employed to depict the metabolic heat emitted from the outermost layer of the skin, yielding medical thermographs of the breast. Cancerous cells induce vasodilation during their initial growth phases, leading to angiogenesis that elevates local temperature prior to tumour development^9^. Thus, breast lesions can modify the skin temperature profile even in their initial stages^10^. Several studies have shown that thermal infrared imaging, also known as thermography, can yield substantial early screening outcomes regarding breast density^11^. This is particularly applicable to women with dense breast tissue. Dynamic thermography is considerably less expensive than MRI and mammography^5^. Additionally, this diagnostic process is non-invasive, safe, and does not produce any ionising radiation or cause harm or pain to patients. Therefore, over the past ten years, researchers have paid close attention to breast thermography for early breast cancer detection^8^.

The recent development of artificial intelligence (AI) methodologies, notably deep learning and machine learning initiated a transformation epoch in medical diagnosis, particularly within medical image processing. These breakthroughs in technology possess the capacity to surpass the constraints of conventional screening techniques, introducing a new era of accuracy and efficacy in medical diagnostics. In the past few years, computer-aided diagnosis and detection (CAD) systems employing deep learning algorithms have attained significant success in diagnosing a range of cancers, including skin cancer^12–16^, cervical cancer^17–19^, leukemia^20^, gastrointestinal related cancer^21–25^, eye diseases^26,27^, and breast cancer^28,29^. CAD systems provide substantial assistance to clinicians by facilitating expedited and autonomous diagnoses, particularly for ailments like breast cancer, while also aiding in detection, monitoring, and reporting procedures. Convolutional neural networks (CNNs), acknowledged as a prevalent deep learning architecture for image analysis, have demonstrated significant effectiveness in medical image tasks, including the identification of breast cancer from thermogram images^11,30–32^. The efficacy of CNNs is primarily attributable to their convolutional functions and the deepness of their layers, where deeper architectures provide enhanced parameter capacity but also introduce challenges, including greater computational cost and increased chances of overfitting^33^.

While there are breakthroughs in the early identification of breast cancer by the methods in different previous studies, there are still several shortcomings and research gaps. For instance, although the CAD systems proposed in the previous works have achieved very good accuracy, the models have often been built on a specific dataset like DMR-IR, and hence they cannot be generalized to other datasets. The use of complex segmentation algorithms has been mainly dominant in previous breast cancer detection studies, which in turn has increased the computational complexity of CAD systems. Moreover, these studies often applied more than one preprocessing step and thus became more cumbersome to evaluate, with no clear impact on the results. Despite some of the research works integrating clinical data with thermal imaging, they were not able to prove that the accuracy of classifying the disease has improved. Furthermore, numerous studies have concentrated on developing tailored CNNs instead of exploiting the benefits of transfer learning and pre-trained CNNs, which can substantially enhance performance with diminished training data and lower computational demands. Moreover, almost all of them depended on individual CNNs, whereas using CNNs with varying architectures for classification would enhance performance. In addition, most of them did not employ a feature selection approach to select features and reduce feature dimensionality thus lowering classification complexity. This highlights shortcomings in the literature concerning the necessity for more efficient methodologies that streamline the CAD process while optimising the utilisation of existing knowledge via transfer learning. This study seeks to overcome these limitations by introducing an innovative CAD methodology that removes preprocessing and segmentation challenges while effectively employing transfer learning, multiple CNNs, and feature transformation and selection approaches to enhance breast cancer detection from thermal images.

The novelty and contributions of the study are summerised as follows:

- Implementing an innovative CAD methodology that obviates intricate segmentation and preprocessing procedures.
- Investigates the capacity of thermal imaging to distinguish normal and abnormal breast tissues as well as benign versus malignant breast cancer.
- Using transfer learning with various pre-trained CNN architectures to enhance breast cancer detection from thermal images.
- Optimising the diagnostic procedure by eliminating superfluous processing steps, thereby decreasing computational complexity.
- Employing a feature transformation technique including non-negative matrix factorisation (NNMF) to reduce dimensionality and extract time-frequency features.
- Applying the Relief-F feature selection approach to select important features, thus achieving a further reduction in complexity.
- Validated the proposed CAD on two datasets to examine Thermo-CAD’s capacity to differentiate between normal/abnormal breast cancer, as well as malignant and benign tumors.

## Literature review

The literature indicates that the majority of prior studies deployed thermogram images from various domains, excluding breast cancer, or employed AI techniques alongside other medical examinations in the context of breast cancer. A minority used both AI methodologies and thermographic images for breast cancer detection. Among these previous works, the study^34^ proposed a CAD based on a U-Net model to autonomously identify and delineate the breast region from the surrounding body, which served as interference for the breast cancer detection model. After that, a specially designed CNN was created and learned to distinguish among normal and abnormal breast thermograms. The proposed system was assessed using real data from the benchmark DMR-IR database, attaining over 99% accuracy. Whereas the research^35^ presented the Enhanced Deep Learning-based Convolutional Neural Network (EDCNN) for the generation of heatmaps from 2D thermal breast images. Those heatmaps offered discernible parameters for evaluating breast vascularity. A classifier was additionally proposed to predict the likelihood of breast cancer based on these parameters. Fuzzy C-means clustering efficiently delineated the area around the breast to enhance accuracy. Temperature profiles were employed to assess segmentation, revealing significant peaks as indicators of regions of interest (ROI), implying potential tumour presence. The EDCNN models, trained on the DMR dataset images, exhibited impressive performance, attaining 96.8% accuracy and 93.7% specificity, outperforming alternative methods.

This study^32^ proposed four unique methodologies for processing thermographic images to improve breast cancer diagnosis within deep learning structures, either independently or in conjunction with evolutionary algorithms. One method entailed disaggregating thermographic images into red, green, and blue channels, with each channel processed by a distinct CNN. An evolutionary algorithm was utilised to allocate differential weights to each network’s output according to performance, thereby optimising the final diagnosis and circumventing mere averaging. Furthermore, two methodologies established a dynamic delineation of low, medium, and high-temperature ranges, customised to the temperature fluctuations among patients, terminating in hybrid models that utilised distinct temperature segments. Finally, a holistic methodology was established wherein complete thermographic images were directly fed into a CNN. The results indicated that the optimal model separated thermographies by temperature spectrum, achieving an accuracy of around 94%.

The research^31^ investigated the efficacy of machine learning methodologies, particularly Bayesian networks integrated with CNNs, to enhance early-stage breast cancer diagnosis. The authors created two systems. The study demonstrated that the first system, which combined thermagrams analysed via explainable artificial intelligence (XAI) with clinical data, achieved an accuracy of 84.07%. Conversely, the second, which used a CNN model, attained an accuracy of 90.93%. A CAD system was created^30^ utilising transfer learning to automatically identify and diagnose possible breast cancer regions. The pre-trained VGG16 model, trained with thermal images from the DMR-IR database, was refined using data augmentation and normalisation methods to enhance accuracy. Although VGG16 yielded remarkable outcomes, alternative techniques such as Support Vector Machines (SVM), K-Nearest Neighbours (KNN), Decision Trees, and Gradient Boosting were also assessed, underscoring the necessity for customised classification strategies. The proposed model achieved remarkable performance, demonstrating a 99.4% accuracy rate, 100% sensitivity, 97.5% specificity, 99% recall, 98.9% precision, 99.8% F1-score, and an AUC-ROC of 99.8%, surpassing earlier breast cancer detection models.

A novel, cost-effective deep learning model, termed LC-SCS, was proposed^36^ for breast cancer classification utilising thermal images based on Sharpened Cosine Similarity. The authors employed pre-trained models: ResNet-101, VGG-16, Inception-V3, ResNet-50, VGG-19, and Xception. The proposed LC-SCS model attained a notable accuracy of 94% on the DMR-IR dataset, falling only 4% short of the superior VGG-19 model while ensuring minimal computational expense. It attained recall and precision scores of 95%, accompanied by an F1 score of 94%. A comprehensible CAD system for breast cancer detection was created^37^, employing XAI and an innovative multi-objective optimisation. Handcrafted techniques were exploited and model decisions were evaluated using Shapley additive explanations (SHAP) on the DMR-IR dataset. Performance improved significantly, with HSMO attaining 98.27% accuracy and a 98.15% F1-score which is better than that achieved by the HOG features by 25.78%. While the study^38^ proposed a CAD for breast cancer detection using thermograms. The study employed several preprocessing steps and applied morphological operation and objected oriented segmentation to segment breast region. Then it constructed a customized CNN to extract features and classify images into healthy and sick.

A lightweight model was developed to identify abnormalities in breast thermograms utilising a deep learning framework followed by a feature selection process^39^. The study utilised SqueezeNet, originally trained on ImageNet, which was subsequently fine-tuned to obtain attributes from tthermogram photos. Researchers employed an innovative hybrid optimisation technique to diminish the overall dimension of the resultant feature vectors. This approach integrated a Genetic Algorithm (GA) with the Grey Wolf Optimiser (GWO), employing chaotic mapping to produce the initial genetic population. The model attained exceptional accuracy in differentiating malignant from healthy breasts, achieving 100% accuracy on the test set of the DMR-IR dataset while using merely 3% of the features extracted by SqueezeNet. The study^40^ proposed a novel approach employing image analysis and machine learning techniques. Thermagram photos were acquired and then augmented using top-hat and bottom-hat transforms during the pre-processing phase. The ROI extraction technique was applied to delineate the breast region while excluding the neck and axillary areas, with structural anomalies eliminated through morphological operations. Statistical, geometrical, and intensity features were derived from the processed images, while texture features were acquired in both spatial and curvelet domains employing a Gray-Level Co-Occurrence matrix. Curvelet wrapping was succeeded by GLCM for the extraction of texture features. Sixteen features were employed to classify the thermal images, with cubic SVM attaining the highest accuracy of 93.3%. The research^41^ relied on a segmentation technique that integrates the curvature function k with the gradient vector flow, and for classification, it proposed a CNN operating the segmented breast reaching an accuracy of 100%.

A novel method^42^ was introduced for the early detection of breast cancer by integrating thermal images from various perspectives with personal and clinical information. A multi-input classification model was developed to utilise the benefits of CNNs for image analysis. Initially, model architectures were examined solely through thermal imagery. Clinical data were subsequently incorporated as an additional branch to each structure to augment performance. The research introduced a breast cancer detection technique utilising multi-view thermograms that were pre-processed with anisotropic diffusion and segmented through level-set methods. Essential texture characteristics were extracted and minimised through statistical tests and kernel principal component analysis (PCA), subsequently integrated across views to create a composite feature vector. The classification step used an SVM classifier which attained 96% accuracy, 100% sensitivity, and 92% specificity. Similarly, a novel CADx system^43^ was designed employing deep learning methodologies trained with thermograms and clinical data for the detection of breast cancer. The methodology involved extracting ROI from images and employing transfer learning to train three distinct models. Findings demonstrated that multi-input models surpassed single-input models, attaining an overall accuracy of 90.48%, and a sensitivity of 93.33%.

This study focuses on the construction and integration of a comprehensive, streamlined, and computationally effective pipeline for breast cancer detection utilizing thermal imaging, addressing various challenges noted in the existing literature.

Initially, Thermo-CAD, in contrast to numerous prior studies that depend extensively on intricate and multi-phase preprocessing or segmentation techniques, completely obviates the necessity for these procedures. This represents a notable divergence from conventional methodologies, which frequently elevate computational expenses and system intricacy without assured enhancements in precision. The proposed framework streamlines the diagnostic process by directly inputting resized images into the system, enhancing its practicality and scalability, particularly in low-resource clinical environments.

Secondly, the implementation of transfer learning utilizing various pre-trained CNN architectures, namely Inception, ResNet-101, and InceptionResNet aimed at acquiring diverse and complementary feature representations. Most previous studies utilize either customized CNNs or a singular pre-trained model. Utilizing a multi-CNN ensemble, Thermo-CAD derives a more comprehensive array of deep features, enhancing the system’s robustness, especially in contexts with constrained training data like thermographic imaging.

Thirdly, the incorporation of NNMF as a feature transformation method, succeeded by Relief-F feature selection, signifies an innovative sequential application of these techniques in thermographic breast cancer detection. NNMF diminishes feature dimensionality and improves interpretability through non-negativity constraints, whereas Relief-F further optimizes the feature space by preserving only the most discriminative attributes. The integration of these two steps markedly diminishes classification complexity and improves model efficacy.

Furthermore, the proposed framework was thoroughly validated on two distinct publicly available datasets, one of which has not been previously utilized for deep learning applications in breast thermography. Thermo-CAD’s capacity to generalize to this new dataset, despite not attaining perfect accuracy, illustrates the model’s wider applicability and indicates opportunities for future enhancement.

## Materials and methods

### Datasets

**Fig. 1:**
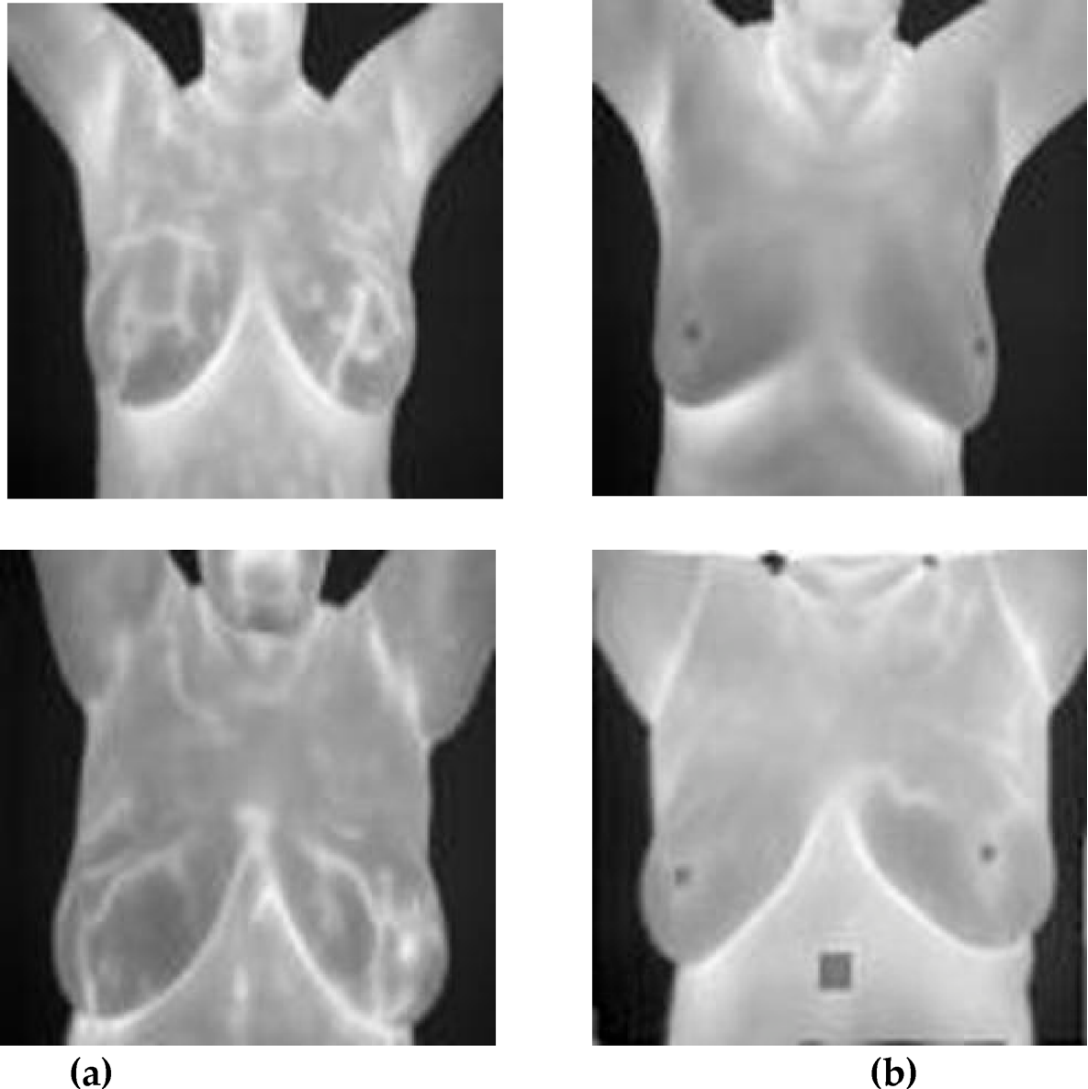
Thermogram Images of the breast DMR-IR dataset; (**a**) abnormal, (**b**) normal.

The proposed CAD is assessed using two datasets. The first one is a benchmark database of the database for mastology research infrared images (DMR-IR)^44^. This database is established by aggregating infrared images from the Hospital of UFF University and is publicly disseminated with the endorsement of the ethics committee, requiring signed consent from each patient. This research utilised a collection of 1000 frontal thermogram images, obtained with a FLIR SC-620 IR camera at a resolution of 640 × 480 pixels from this database, comprising 500 normal and 500 abnormal cases. These images depict breasts of diverse shapes and sizes (refer to Fig. 1).

**Fig. 2:**
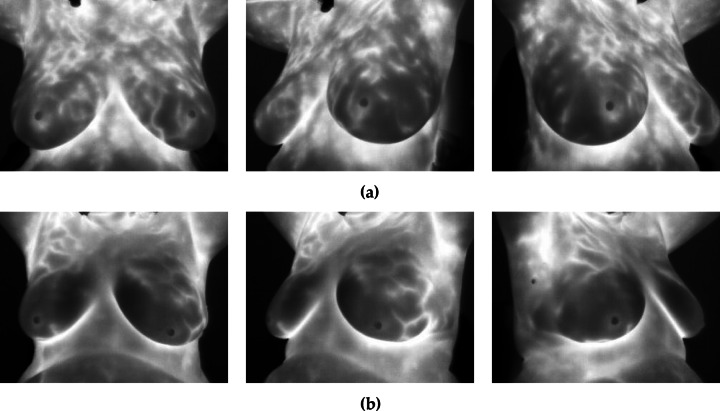
TExample of thermogram images of the breast in the second dataset; (**a**) benign, (**b**) malignant.

The second dataset consists of thermographic images of the female chest^45^. The dataset comprises images of 119 women, aged 18 to 81, with each participant depicted in three perspectives: anterior, left oblique, and right oblique. The diagnoses for these patients reveal that 84 had benign breast pathologies and 35 had malignant ones, while data for patients with normal breast pathologies were excluded. The images were obtained at San Juan de Dios Hospital – Consultorio Rosado in Cali, Colombia, under controlled conditions in accordance with the American Academy of Thermology (AAT) protocol. Imaging occurred in a medical office (3.20 m x 4.14 m x 2.40 m) maintained at 22–24 °C with 45–50% relative humidity, devoid of artificial lighting. A FLIR A300 camera was employed, situated 1 m from the patient and 88 cm above the floor, with patients remaining at rest for 8 min before imaging. The dataset comprises thermographic data and patient information, including age, weight, height, body temperature, and comprehensive pathology reports. Figure 2 displays examples from this dataset.

### Proposed Thermo-CAD framework

**Fig. 3:**
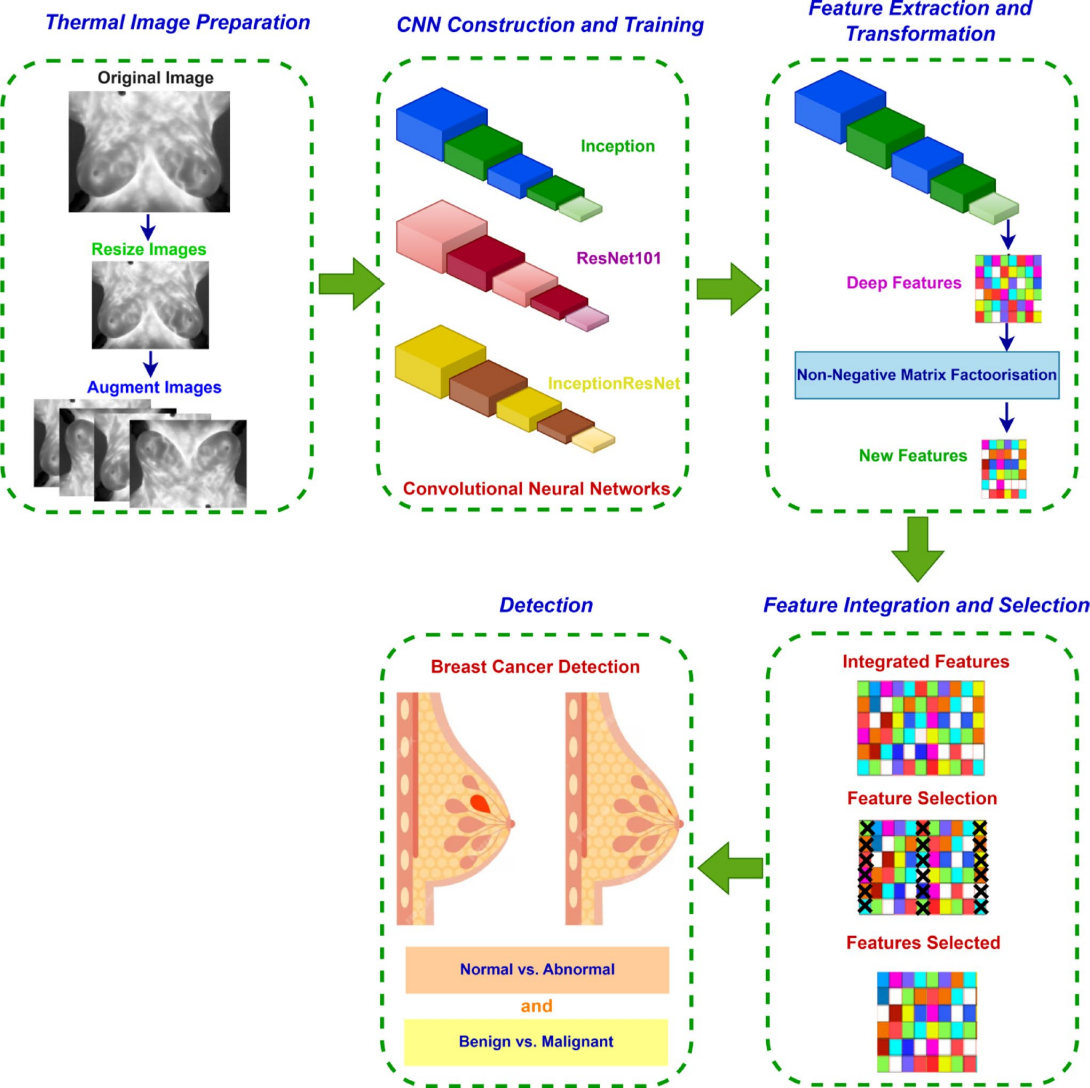
Stages of the proposed Thermo-CAD framework.

The proposed Thermo-CAD framework comprises five consecutive stages: thermal image preparation, CNN construction and training, feature extraction and transformation, feature integration and selection, and detection. During the preliminary image preparation phase, photos are resized and augmented to improve diversity and resilience. During the following stage, three pre-trained CNN models—ResNet-101, Inception, and InceptionResNet—are modified and optimised for thermogram images. Next, upon completion of the training of the CNNs, deep features are obtained from each model. Afterward, the features are decreased in dimensionality through a NNMF transformation approach. Subsequently, the features extracted from the three CNNs are combined, after which the Relief-F algorithm is employed to further diminish dimensionality by selecting the most discriminative features. Ultimately, various SVM classifiers are employed to categorise the images as normal or abnormal, or as benign or malignant. Figure 3 depicts the workflow of the Thermo-CAD framework.

#### Image preparation

The thermographic images from both datasets are preprocessed to conform to the input sizes needed by the three CNN models, specifically 224 × 224 × 3. This scaling guarantees that each image matches the input requirements of the CNNs, thereby preserving uniformity within the dataset and enhancing compatibility with the network structure. Subsequent to this modification, each dataset is partitioned into separate training and testing subsets, with 70% of the data designated for training and 30% reserved for testing, thereby creating a standardised framework for model assessment. To enhance model robustness and reduce the likelihood of overfitting, the training data is subjected to comprehensive augmentation employing a variety of techniques. Those augmentation techniques substantially enlarge the dataset by incorporating variations in the photos, including rotations, flips, and scaling modifications. This method improves the model’s generalisation capability and augments the training process by presenting the model with a broader spectrum of image scenarios. Table 1 delineates the particular information about the augmentation processes employed, offering a thorough overview of the approaches utilised to enhance model performance. It is worth mentioning that, the data was split prior to any data augmentation to eliminate any possibility of data leakage. Specifically, both datasets were split into training and test subsets (70%/30%) before applying augmentation solely to the training subsets. This way, there would be no augmented versions of the same image present in both training and test datasets, guaranteeing the evaluation process was done without any breaches or data leakage during training.

**Table 1:** Augmentation processes applied to both datasets.

Augmentation Methodology	Ranges
Flipping vertically and horizontally	− 55 to 55
Rotational in both x and y orientations	− 90 to 90
Scaling	0.1 to 2.5
Shear vertically	− 65 to 65

#### CNNs construction and training

This study employs transfer learning leveraging three CNNs that have been pre-trained on the ImageNet dataset^46^. The considered architectures are Inception^47^, ResNet101^48^, and InceptionResNet^49^ because of their complementary strengths: Inception captures features concurrently at multiple scales with its parallel convolutional filters, while ResNet-101 mitigates the vanishing gradient problem with its very deep residual learning framework. Whilst Inception-ResNet combines both advantages: better feature extraction capabilities and improved gradient flow. The architecture of each CNN is changed to have two fully connected layers, being that amount needed for binary classification of breast cancer detection in the datasets employed in this study. Thereafter, hyperparameter fine-tuning is accomplished, according to the details within the experimental setup section. Afterward, the retraining of the networks begins, each of them is trained independently with the thermogram images. Because this is independent training, it means that the exploitation of the peculiar strengths of each architecture can be achieved while still preserving integrity in their respective learning processes.

#### Feature extraction and transformation

Feature extraction constitutes a crucial element of the Thermo-CAD architecture and is essential for its capacity to accurately identify breast cancer. This study employed a transfer learning method for feature extraction using three recognized, pre-trained CNN structures: Inception, ResNet-101, and InceptionResNet. Each of these models, initially trained on the ImageNet dataset, was chosen for its mutual advantages in capturing varied levels of spatial and semantic information. Inception effectively extracts multi-scale features via parallel convolutional filters, ResNet-101 utilizes profound residual connections to mitigate vanishing gradient problems, and InceptionResNet integrates both deep and wide representations to improve discriminative power.

In the feature extraction phase, thermal photographs from both datasets; DMR-IR (normal vs. abnormal classification) and the second dataset (benign vs. malignant classification), were handled through these networks following suitable resizing to satisfy input size specifications. High-level deep features were derived from the final pooling layer of each CNN, providing a concise representation of the most distinguishing spatial and contextual patterns in the input images. Such layers have been identified for encoding abstract and useful attributes essential for classification tasks, incorporating variances in temperature distribution and thermal fluctuations indicating abnormalities in the breast or malignancies.

The dimensions of the extracted feature vectors differed among the three CNNs: Inception and ResNet101 both yielded 2048-dimensional features, whereas InceptionResNet yielded 1536-dimensional features. Such high-dimensional vectors encompass intricate thermal patterns that are not readily apparent through manual examination, yet are very valuable for automated classification. Nevertheless, due to the extensive array of features, the direct application of these raw vectors may result in overfitting, heightened computational complexity, and diminished model interpretability. Consequently, a two-step post-processing method was used to refine the feature space: feature transformation using Non-Negative Matrix Factorization (NNMF) and Relief-F-based feature selection.

Feature transformation and dimensionality reduction strategies are vital in mitigating overfitting by removing unnecessary and redundant features. The NNMF method is distinguished among these techniques for its ability to analyse huge datasets and derive significant representations while minimising dimensionality. The application of feature reduction and transformation techniques enables the elimination of non-essential features, resulting in solutions with diminished computational complexity. Although accuracy is crucial in AI-driven medical diagnostic applications, the reliability of the system and computational effectiveness also require significant attention.

To achieve this goal, the NNMF feature transformation and reduction approach is applied to deep features extracted from each CNN. NNMF is a sophisticated computational technique that breaks down a non-negative matrix *V* into the product of two lower-rank non-negative matrices *W* and *H*, such that *V ≈ WH*^50^. This method is especially beneficial for dimensionality reduction and feature extraction, as it produces comprehensible, parts-based illustrations that represent the input data^51^. In contrast to other matrix factorisation techniques like PCA, NNMF imposes non-negativity restrictions, guaranteeing that all elements in the resultant matrices are non-negative^52^. This restriction renders NNMF particularly appropriate for examining data where negative values are nonsensical, such as pixel intensities in photos.

The mathematical representation of NNMF may be articulated in the following manner: For a matrix *V* $\in$ *ℝ^(m×n)*, NNMF identifies non-negative matrices W $\in$ ℝ^(m×k) and *H* $\in$ *ℝ^(k×n)* that minimise the reconstruction error between *V* and the product *WH*^53^. The optimisation problem may be expressed as:

1 $$minimize{\mkern 1mu} \left| {\left| {V{\mkern 1mu} - {\mkern 1mu} WH} \right|} \right|^{2} {\mkern 1mu} subject{\mkern 1mu} to{\mkern 1mu} W,{\mkern 1mu} H{\mkern 1mu} \ge {\mkern 1mu} 0$$

where ||·|| signifies the Frobenius norm, and k is generally selected to be less than both m and n, yielding a reduced version of the original data^54^.

- *W*: An m × r matrix representing a basis for the data, where each column corresponds to a part or component.
- *H*: An r × n matrix representing the coefficients or weights of the basis in *W*.

The non-negativity condition yields numerous beneficial properties. Initially, it enhances the interpretation of results by representing each feature as an additive aggregation of components, which corresponds to an individual’s intuitive comprehension of component-based systems. Secondly, the resultant sparse representation frequently encapsulates the fundamental patterns in the data more efficiently than conventional methods^55^. This sparsity enhances generalisation and can uncover latent structures that may be concealed in the original high-dimensional space.

#### Feature integration and selection

The diminished features obtained from the three CNNs are integrated via concatenative fusion. This merging process produces a higher-dimensional feature space, requiring further feature selection. The method of selecting features includes various methods aimed at identifying important attributes and eliminating redundant as well as unnecessary variables, thus simplifying classification tasks and reducing the risk of overfitting^14^. Features are chosen at the current phase using Relief-F feature selection. Relief-F^56^ is an advanced algorithm developed for feature selection in classification tasks. The algorithm fundamentally assesses the importance of variables by analysing their efficacy in differentiating between comparable data points. The procedure allocates weights to variables according to their capacity to distinguish between very similar instances within the dataset.

The algorithm functions by initially choosing a data point at random, referred to as Ri. It subsequently identifies two essential adjacent points: the “nearest hit” (*T*), representing the closest data point of the same class as Ri, and the “nearest miss” (M), denoting the closest point from the opposing class. This process allows Relief-F to evaluate the efficacy of each feature in differentiating between similar instances from distinct classes. The weight calculations for every attribute (*We(Fe)*) are ascertained by examining the variance in feature values among these meticulously chosen points. This method enables Relief-F to accurately measure the discriminative strength of every parameter in the classification task as shown in Eq. (2).

2 $$We\left(Fe\right)\triangleq We\left(Fe\right)-\frac{diff\left(Fe, {R}_{i},T\right)}{m}+\frac{diff\left(Fe, {R}_{i},M\right)}{m}$$

The functions *diff(F*,* Ri*,* T)* and *diff(F*,* Ri*,* M)* denote two essential metrics: the proximity of an instance to other instances within its class, and its proximity to examples from different classes, respectively. The user-defined parameter *m* indicates the entire amount of iterations to execute.

#### Detection

The detection phase of the proposed Thermo-CAD model is conducted in a pair of phases. In the early phase, features derived from CNNs learned on the initial DMR-IR dataset are employed to distinguish between normal and abnormal breast tissues. The next phase leverages deep features extracted from CNNs trained on the second dataset to categorise abnormal cases as benign or malignant. To achieve these goals, five Support SVM classifiers using different kernel functions are employed: Linear (LSVM), Quadratic (QSVM), Cubic (CSVM), Medium Gaussian (MGSVM), and Coarse Gaussian (CGSVM). The assessment of these models is executed using a five-fold cross-validation method.

## Experimental setup and performance metrics

This section outlines the effectiveness metrics used to evaluate the efficiency of the suggested Thermo-CAD model, along with optimised hyperparameter settings. Each CNN is subjected to rigorous hyperparameter fine-tuning. Table 2 delineates the complete hyperparameter values, with a mini-batch size fixed at 5 and training executed for a total of 100 epochs. The learning rate is set at 0.003, with a validation frequency of 140 for the first dataset and 50 for the second dataset. All other parameters maintain their default values. The networks adopt Stochastic Gradient Descent with Momentum (SGDM) as the optimisation algorithm during the learning process.

**Table 2:** Hyperparameter values that are fine-tuned for the cnns.

Hyperparameters	Values
Mini batch	5
Epochs	100
Learning rate	0.003
Validation frequency	140 50
First IDM-IR Dataset	
Second Dataset	

The choice to train each CNN model for 100 epochs was determined by a balance of experimental efficiency, convergence characteristics, overfitting mitigation, and adherence to established protocols in transfer learning for medical imaging. Initial experiments revealed that the Inception, ResNet-101, and InceptionResNe CNNs necessitated a moderate quantity of training iterations to proficiently adjust their weights to the thermal imaging domain via transfer learning.

Evaluations of these models revealed that training beyond 100 epochs yielded negligible enhancements in validation accuracy or loss, while heightening the risk of overfitting, especially considering the relatively small dataset sizes. In contrast, training for fewer than 100 epochs frequently resulted in inadequate convergence and diminished classification efficacy. Consequently, 100 epochs were chosen as a reasonable epoch count that enabled the networks to acquire domain-specific characteristics without overtraining that could impair generalization. The 100-epoch training procedure guaranteed that the feature representations derived from these networks were adequately refined for subsequent transformation and classification tasks, while avoiding excessive computational expense or overfitting.

The measurements of evaluation are essential to evaluating the effectiveness of deep learning methods, providing critical insights into their effective properties. The principal metrics employed for model assessment encompass accuracy, precision, sensitivity, specificity, Matthews Correlation Coefficient (MCC), F₁-score, Receiver Operating Characteristic (ROC) curve, and the Area Under the Curve (AUC). Accuracy measures the proportion of correct predictions to total predictions, offering a thorough evaluation of model performance. Precision assesses the model’s proficiency in accurately identifying positive cases, whereas sensitivity, also known as the True Positive Rate (TPR), measures its effectiveness in detecting all positive instances.

The F₁-score functions as a composite metric of model efficacy by integrating both precision and recall measurements. These measurements are essential for identifying overfitting, optimising model parameters, and guaranteeing robust performance. Specificity, also referred to as the True Negative Rate (TNR), denotes the ratio of accurately identified negative instances to the overall negative observations and acts as the complement to sensitivity. The AUC metric, primarily utilised in binary classification, functions as an assessment tool for the model’s ability to rank instances among various classes. The MCC measures the degree of correlation between predicted and actual classification labels. These metrics are articulated through Eqs. (3–9).

3 $$Sensitivity=\frac{TP}{TP+FN}$$

4 $$Specificity=\frac{TN}{TN+FP}$$

5 $$Precision=\frac{TP}{TP+FP}$$

6 $$MCC=\frac{TP\times TN-FP\times FN}{\sqrt{(TP+FP)(TP+FN)(TN+FP)(TN+FN)}}$$

7 $$F\_1-score=\frac{2\times TP}{\left(2\times TP\right)+FP+FN}$$

8 $$`Accuracy=\frac{TP+TN}{TN+FP+FN+TP}$$

9 $$AUC=\sum TP+ \sum \frac{TN}{P+N}$$

While True Negatives (TN) comprise every case precisely identified as negative (N), True Positives (TP) indicate the total amount of cases properly identified as positive (P). False Negatives (FN) are cases mistakenly categorised as negative when they are, in fact, positive; False Positives (FP) are examples mistakenly identified as positive when they are actually negative.

## Results

This section presents the empirical results of the suggested Thermo-CAD model. The results from the preliminary DMR-IR dataset (initial detection phase), intended to distinguish between normal and abnormal breast tissue, are discussed in the first subsection. This subsection first analyses and discusses the performance of machine learning classifiers trained independently using deep features extracted from each CNN. The outcomes after feature transformation and dimensionality reduction are subsequently presented. Next, the findings following feature integration from all three CNNs and the application of the Relief-F feature selection method are thoroughly explored. The second subsection delineates the results from the second dataset, which focuses on differentiating among the two categories of breast cancer abnormalities, including benign and malignant, during the second detection phase. A similar analytical framework to that deployed in the initial subsection is adopted, ensuring procedural consistency throughout the analysis.

### First dataset results (normal versus abnormal)

This section explains and examines the experimental findings of the presented Thermo-CAD framework, showcasing its efficacy in distinguishing between normal and abnormal breast thermal images. Table 3 displays the performance derived from deep features directly extracted from separate CNN models. As shown in Table 3, InceptionResNet attained the highest accuracy of 99.3% using CSVM, shortly followed by 99.1% with MGSVM. While the Inception model produced accuracies between 95.0% and 98.3%, with both CSVM and QSVM attaining the maximum performance of 98.3%. Whereas ResNet101 demonstrated marginally lower yet commendable results, varying from 95.5 to 98.4%, with CSVM attaining the peak accuracy. The findings demonstrate that InceptionResNet’s structure is exceptionally effective at obtaining discriminative features from thermal photos, while the uniform performance across various SVM variants demonstrates the reliability of the features retrieved.

**Table 3:** SVMs classification accuracy (%) achieved with deep features of each CNN model for the first DMR-IR dataset.

Model	LSVM	QSVM	CSVM	MGSVM	CGSVM
Inception	98.1	98.3	98.3	98.0	95.0
InceptionResNet	98.9	98.9	99.3	99.1	96.3
ResNet101	97.5	97.9	98.4	97.6	95.5

The implementation of NNMF for feature transformation and reduction on the aggregated deep features of the three CNNs, as outlined in Table 4, uncovered variations across various feature dimensions. The accuracy for Inception features reached a maximum of 99.0% with LSVM utilising 50 features, maintaining consistent performance above 98.5% with 20 or more features. The performance was consistent across various feature dimensions, indicating efficient feature reduction while preserving discriminative capability. ResNet-101 exhibited greater performance variability, attaining a peak accuracy of 98.2% with LSVM using 40 features. A distinct trend was observed, demonstrating enhanced performance as the number of features increased from 10 (94.5%) to 40, featuring the significance of preserving adequate feature dimensionality for this design. InceptionResNet exhibited strong performance, attaining 99.3% accuracy with MGSVM at 30 features, and consistently surpassing 98.0% accuracy across various feature dimensions. This stability indicates that InceptionResNet features are especially conducive to dimensionality reduction while maintaining essential classification information.

**Table 4:** First dataset (normal versus abnormal detection task) SVMs classification accuracy (%) achieved with reduced deep features of each CNN model using the NNMF feature transformation and reduction approach.

	LSVM	QSVM	CSVM	MGSVM	CGSVM
*Inception Features*					
10	96.6	97.6	97.5	97.2	95.0
20	98.7	98.6	98.5	98.7	97.7
30	98.8	98.9	98.8	98.8	98.3
40	98.7	98.7	98.6	98.6	98.5
50	99.0	98.9	98.9	98.6	98.0
100	98.7	98.7	98.5	98.7	97.9
*ResNet-101 Features*					
10	94.5	97.3	96.0	97.2	93.3
20	96.1	96.9	96.9	97.6	95.9
30	96.8	97.1	96.7	97.3	95.5
40	98.2	97.9	97.7	97.4	97.2
50	97.8	97.4	97.6	97.5	97.6
100	97.7	97.4	97.6	97.6	97.3
*InceptionResNet Features*					
10	97.4	98.4	98.4	98.4	96.3
20	97.9	98.6	98.9	96.7	96.6
30	98.8	98.8	98.8	99.3	97.8
40	98.2	98.6	98.3	98.9	97.4
50	98.8	98.9	99.0	98.8	98.3
100	98.7	98.6	98.6	98.4	98.8

**Fig. 4:**
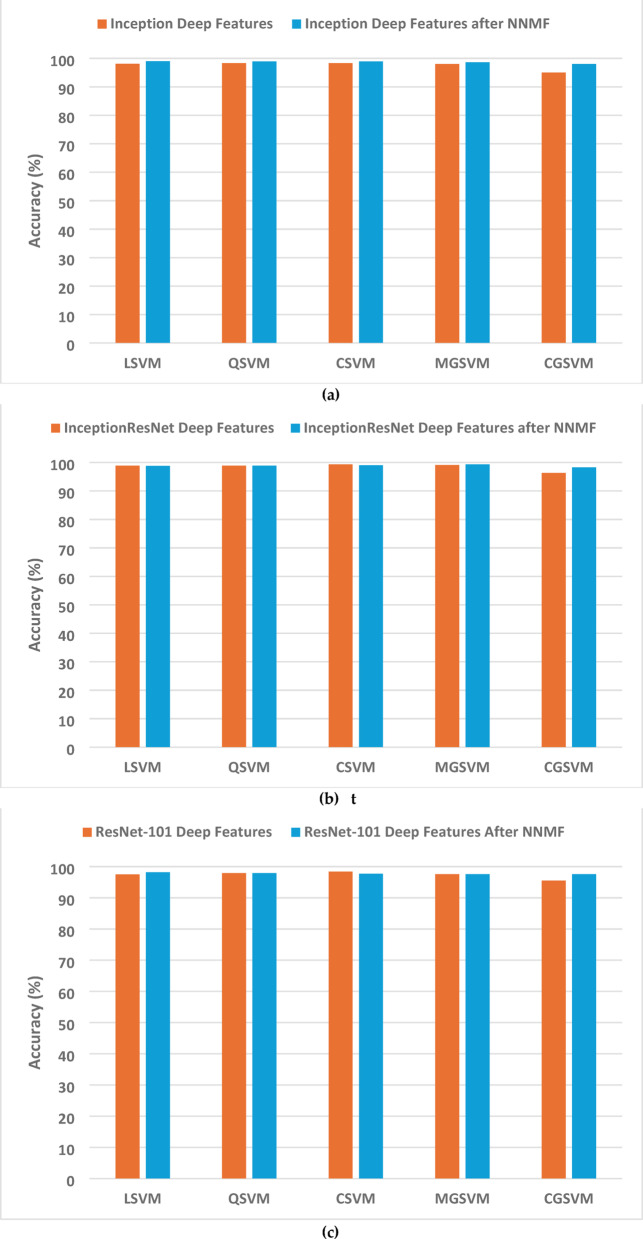
Classification accuracy comparison before and after applying the NNMF method for the three CNNs trained on the first dataset (normal versus abnormal detection task): (**a**) Inception, (**b**) ResNet-101, and (**c**) InceptionResNet.

Table 5 displays the most notable results derived from the application of Relief-F feature selection to the integrated features from all three CNNs. Optimal classification (100% accuracy) was attained across various configurations: with QSVM, CSVM, and MGSVM employing 70–100 features. The performance demonstrated significant resilience to feature reduction, sustaining high accuracy despite reduced feature sets. The accuracy, utilising only 10 chosen features, remained remarkably high (98.3–99.2%). The performance exhibited steady enhancement with an increasing number of features: 20 features resulted in 99.1–99.4%, 30 features attained 99.1–99.8%, and 60 features consistently delivered accuracies exceeding 99.7%. This incremental enhancement resulted in near-perfect or outstanding classification with 80 or greater attributes across all SVM variants. The classification accuracy with confidence intervals (CI) for the SVM classifiers trained with the selected features which achieved the highest performance after applying the Releif-F feature selection method Fig. 4.

**Table 5:** First dataset (normal versus abnormal detection task) SVMs classification accuracy (%) SVMs attained with the chosen attributes subsequent to the application of Relief-F feature selection on the aggregated deep features of the three deep models.

Model	LSVM	QSVM	CSVM	MGSVM	CGSVM
10	99.0	99.2	99.0	99.0	98.3
20	99.4	99.3	99.3	99.3	99.1
30	99.5	99.5	99.5	99.8	99.1
40	99.0	99.2	99.0	99.0	98.3
50	99.3	99.6	99.7	99.7	99.3
60	99.7	99.8	99.8	99.9	99.7
70	99.8	99.7	99.9	100	99.7
80	99.9	99.9	99.9	100	99.8
90	99.8	99.9	99.9	100	99.9
100	99.9	99.9	100	100	99.9

**Fig. 5:**
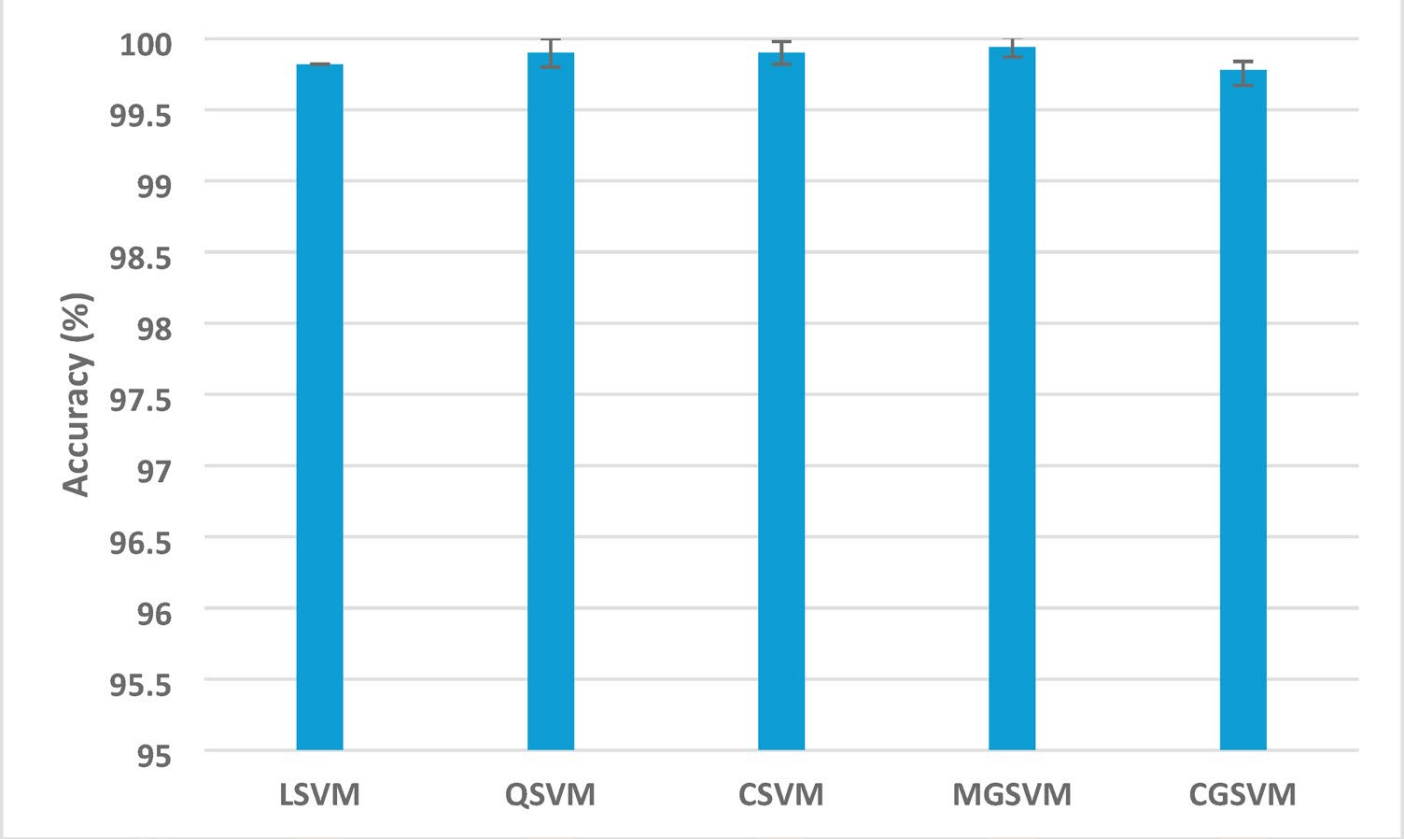
Classification accuracy with confidence intervals (CI) for the SVM classifiers trained with the selected features which achieved the highest performance after applying the Releif-F feature selection method.

The classification accuracy, along with confidence intervals (CI), for the SVM classifiers developed using the chosen features, demonstrating their peak performance after the implementation of the Relief-F feature selection method—are presented in Fig. 5. The findings reveal that all classifiers exhibited remarkably high accuracy, with mean scores spanning from 99.78 (CGSVM) to 99.94% (MGSVM). The CIs for all classifiers are narrow, indicating the consistency and reliability of the model’s performance. MGSVM attained the highest mean accuracy, reaching an upper bound of 100%, thereby reaffirming the robustness of the Thermo-CAD framework in differentiating between normal and abnormal breast thermal photographs with outstanding accuracy.

Additional evaluation measures are computed for the peak performance achieved in Table 5 and displayed in Table 6. These measures include sensitivity, precision, F1-score, MCC, and specificity. In addition, the confusion matrices for the CSVM and MGSVM as they reached the greatest performance in Table 5 are displayed in Fig. 4. In addition, the ROC curves for the same classifiers are shown in Fig. 5. The AUC is also determined and revealed in Fig. 4. Table 6 displays essential performance metrics for five categories of SVM classifiers, which were trained using selected features following the application of Relief-F feature selection on deep features derived from three CNN architectures. The CSVM and MGSVM classifiers attained optimal performance, achieving a sensitivity, specificity, precision, F1-score, and MCC of 1.000. These findings demonstrate the model’s capacity to attain flawless classification, precisely recognising every case without false positives or false negatives. The performance of the other classifiers—LSVM, QSVM, and CGSVM—was similarly exceptional, with scores ranging from 0.9980 to 0.9990, signifying consistent and robust model efficacy across diverse classification methodologies.

**Table 6:** First dataset (normal versus abnormal detection task) evaluation measures calculated for the SVM classifiers trained with the chosen attributes subsequent to the application of Relief-F feature selection on the aggregated deep features of the three deep models.

Metric	LSVM	QSVM	CSVM	MGSVM	CGSVM
Sensitivity	0.9980	0.9980	1.000	1.000	0.9980
Specificity	1.000	1.000	1.000	1.000	1.000
Precision	1.000	1.000	1.000	1.000	1.000
F1-score	0.9990	0.9990	1.000	1.000	0.9990
MCC	0.9980	0.9980	1.000	1.000	0.9980

**Fig. 6:**
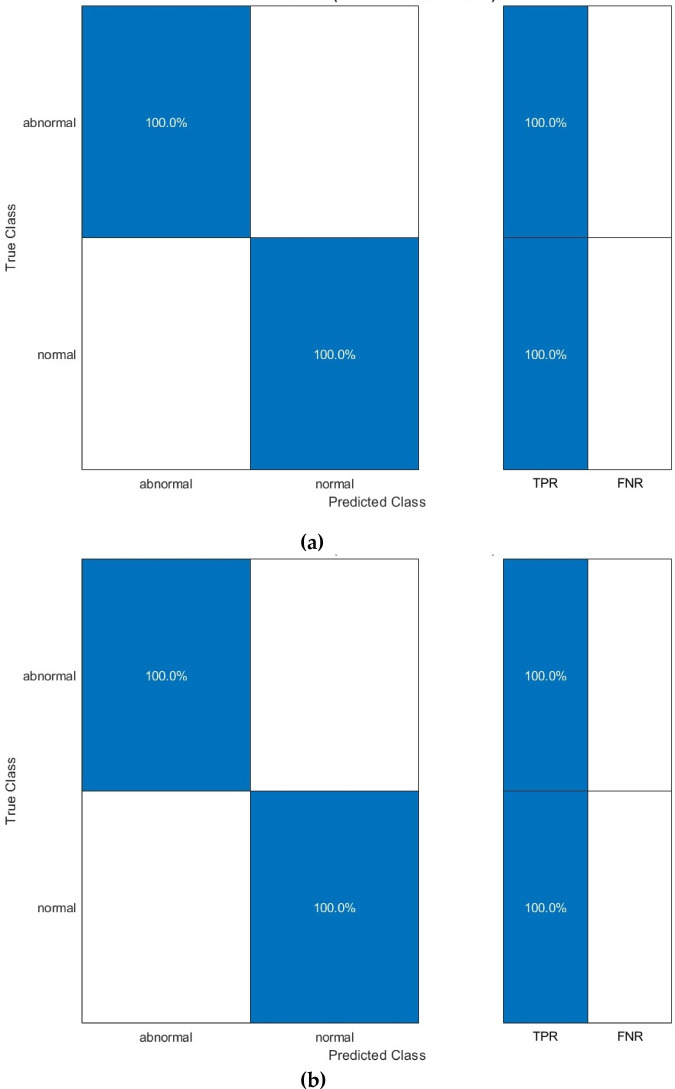

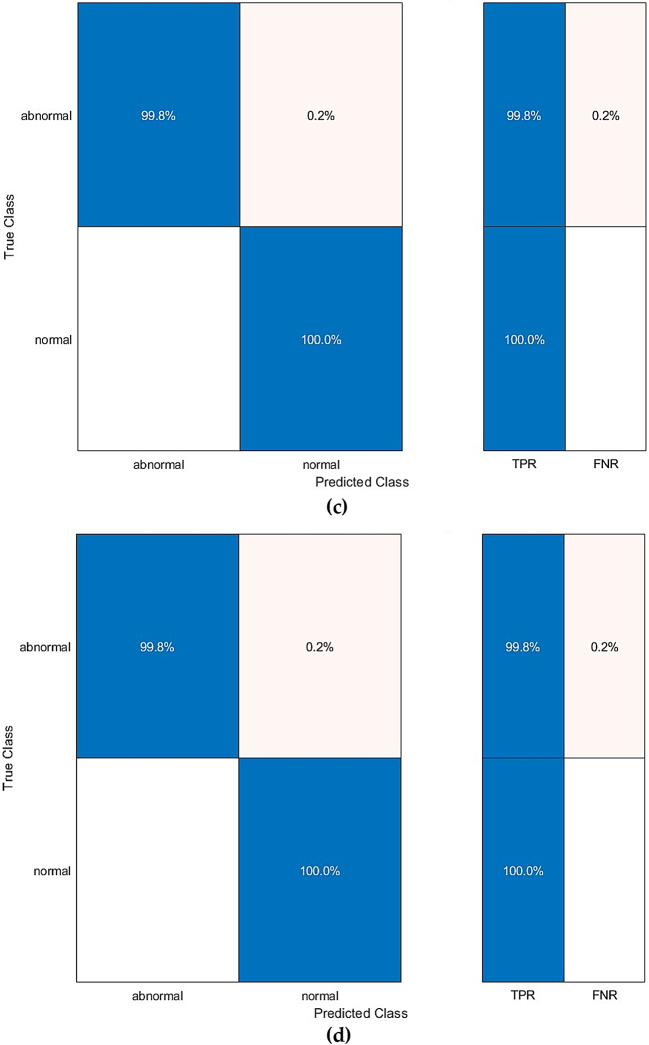
First dataset (normal versus abnormal detection task) confusion matrices for the (**a**) CSVM, (**b**) MGSVM, (**c**) LSVM, and (**d**) QSVM classifiers after the Relief-F feature selection step.

**Fig. 7:**
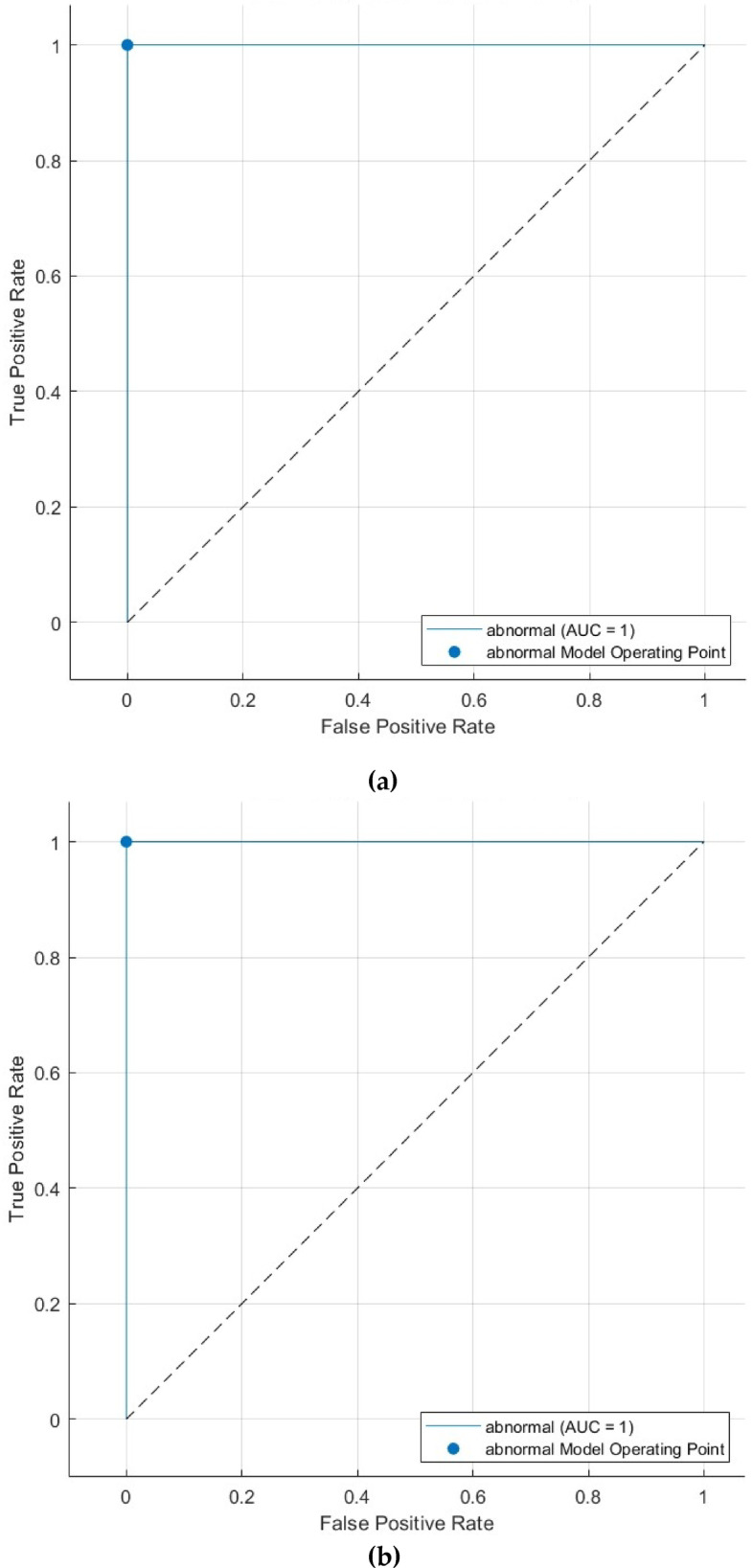

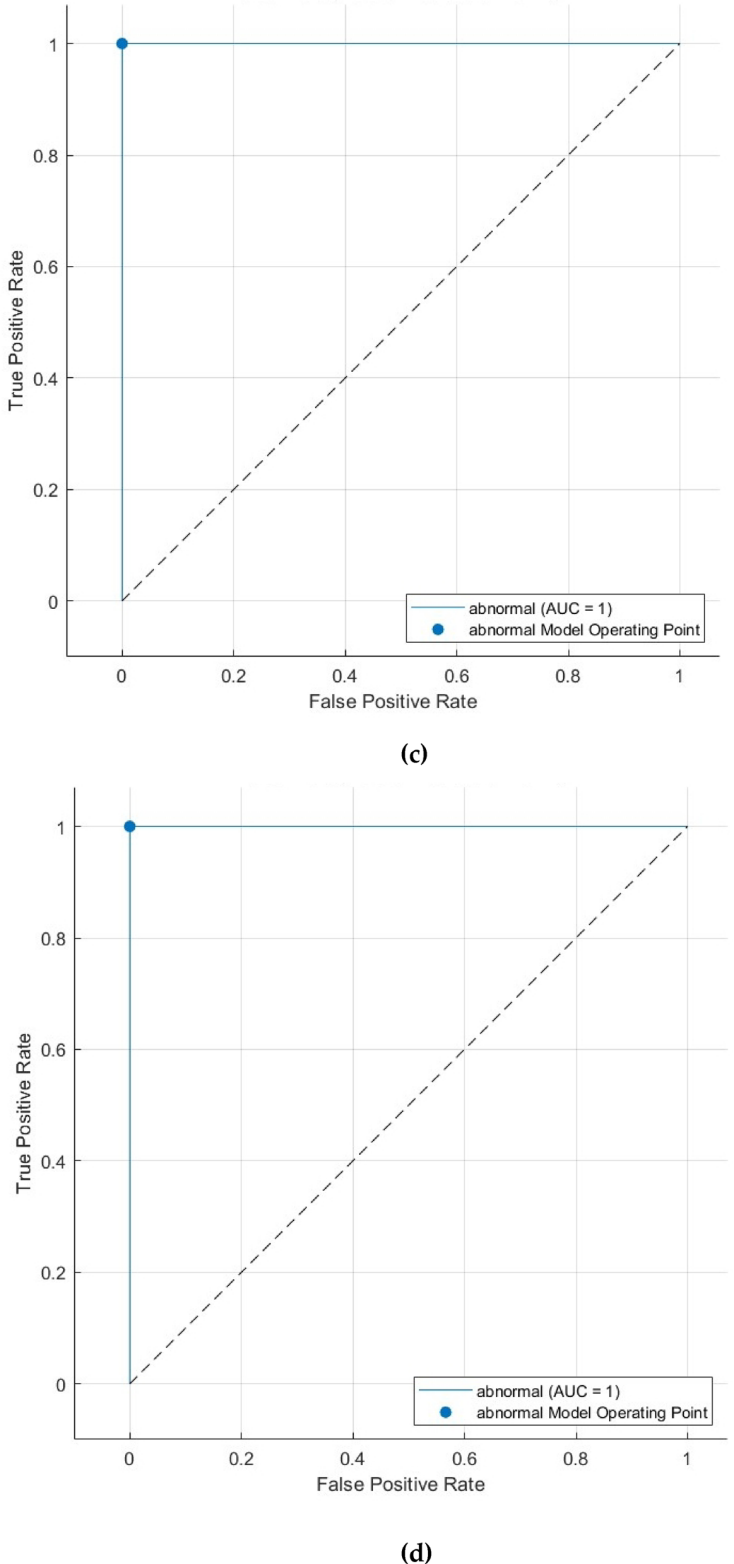
First dataset (normal versus abnormal detection task ROC curves for the (**a**) CSVM, (**b**) MGSVM, (**c**) LSVM, and (**d**) QSVM classifiers after the Relief-F feature selection step.

Figure 7 presents the ROC curves for the CSVM, MGSVM, LSVM, QSVM, and CGSVM classifiers, which additionally show the model’s superior performance. ROC curves exhibit an AUC of 1.000, signifying outstanding sensitivity and specificity. The AUC score indicates that the classifiers exhibit strong performance and consistent accuracy across different decision thresholds, demonstrating robust reliability in classification. An AUC of 1.000 corresponds with the perfect sensitivity and specificity indicated in Table 6, validating the model’s ability to differentiate between normal and abnormal cases at all operating points.

The enhanced efficacy of the integrated feature method (Table 5) relative to distinct CNN features (Table 3) substantiates the hypothesis that features derived from various CNN models encompass complementary dimensions of thermal images. This redundancy yields a more thorough representation for classification, enhancing diagnostic performance. The incremental enhancement in accuracy throughout the pipeline—from individual CNN features to NNMF transformation to Relief-F selection—illustrates the efficacy of a multi-stage feature processing methodology. Every stage enhances overall performance, culminating in the final stage, which attains flawless classification while preserving computational efficiency. Moreover, the efficacy of dimensionality reduction via NNMF and Relief-F feature selection illustrates the potential to preserve high classification accuracy while substantially decreasing computational complexity. This is especially apparent in attaining 100.0% accuracy with MGSVM using only 70 selected features post-Relief-F selection, in contrast to the original high-dimensional feature spaces of the CNNs (2048, 2048, and 1536 features of the Inception, ResNet101, and InceptionResNet, respectively). The decrease in dimensionality has practical consequences for real-time processing and resource-limited settings. In addition, the accomplishment of flawless classification through various feature-classifier combinations highlights the efficacy of the proposed Thermo-CAD framework in tackling the challenges of breast cancer detection from thermal images. These findings collectively affirm Thermo-CAD as a reliable and effective framework for differentiating between normal and abnormal breast thermal images.

### Second dataset results (benign versus malignant)

This section presents a thorough analysis of the Thermo-CAD model’s efficacy across different classifiers and feature extraction techniques. Tables 7, 8 and 9 present the findings for differentiating between benign and malignant breast cancer exploiting diverse deep features derived from three CNN models (Inception, InceptionResNet, and ResNet101), feature transformation via NNMF, and feature selection employing the Relief-F algorithm.

Table 7 displays the outcomes derived from deep features that are obtained from each of the CNN models. It is clear from Table 7 that InceptionResNet demonstrated better results, achieving 77.3% accuracy with the MGSVM classifier. The Inception model attained a peak accuracy of 72.8% using MGSVM, whereas ResNet101 reached a maximum of 72.5% with CSVM. The findings, although outstanding, do not reach the near-perfect classification attained in the normal-abnormal detection task, indicating that the thermal signatures differentiating benign from malignant tumours are more complex and difficult to capture.

**Table 7:** Second dataset (benign versus malignant detection task) SVMs classification accuracy (%) achieved with deep features of each CNN model for the second dataset.

Model	LSVM	QSVM	CSVM	MGSVM	CGSVM
Inception	72.3	68.6	67.5	72.8	70.6
InceptionResNet	75.9	74.5	74.2	77.3	70.0
ResNet101	71.7	72.0	72.5	70.6	70.6

The deployment of NNMF feature transformation, as described in Table 8, demonstrated deeper patterns in how well classification was performed. As seen in Table 8, Inception features achieved a maximum accuracy of 74.8% with LSVM using 10 features. ResNet101 features attained a peak performance of 74.2% with LSVM at 50 features, demonstrating increased variability in performance across varying feature counts. InceptionResNet features exhibited superior performance, attaining 77.3% accuracy with MGSVM utilising 50 features, and consistently surpassing 70% across diverse feature dimensions. The results demonstrate that although NNMF effectively improved classifier performance for the benign-malignant classification task, the optimal feature dimensionality was largely contingent upon the underlying CNN architecture.

**Table 8:** Second dataset (benign versus malignant detection task) SVMs classification accuracy (%) achieved with reduced deep features of each CNN model using the NNMF feature transformation and reduction approach for the second dataset ( benign versus malignant).

		LSVM	QSVM	CSVM	MGSVM	CGSVM
*Inception Features*						
10		74.8	70.0	66.7	74.5	70.6
20		74.2	73.1	68.3	73.4	70.6
30		70.3	64.7	61.9	70.6	70.6
40		73.4	70.6	67.5	72.8	70.6
50		70.9	72.3	73.1	72.5	70.6
100		74.2	72.5	71.1	73.1	70.6
*ResNet-101 Features*						
10		70.6	71.4	63.6	71.7	70.6
20		71.1	71.7	70.9	69.7	70.6
30		70.3	68.3	65.3	70.6	70.3
40		73.1	73.4	69.7	71.7	70.6
50		73.7	74.2	69.5	72.0	70.6
100		70.3	71.4	72.3	71.7	70.3
*InceptionResNet*						
10		75.6	70.9	64.7	73.9	71.7
20		75.6	70.6	67.5	77.0	71.1
30		73.4	66.4	63.9	75.9	70.6
40		76.2	71.7	66.1	75.6	70.9
50		77.0	74.2	73.4	77.3	70.6
100		75.1	71.4	72.3	74.8	70.6

**Fig. 8:**
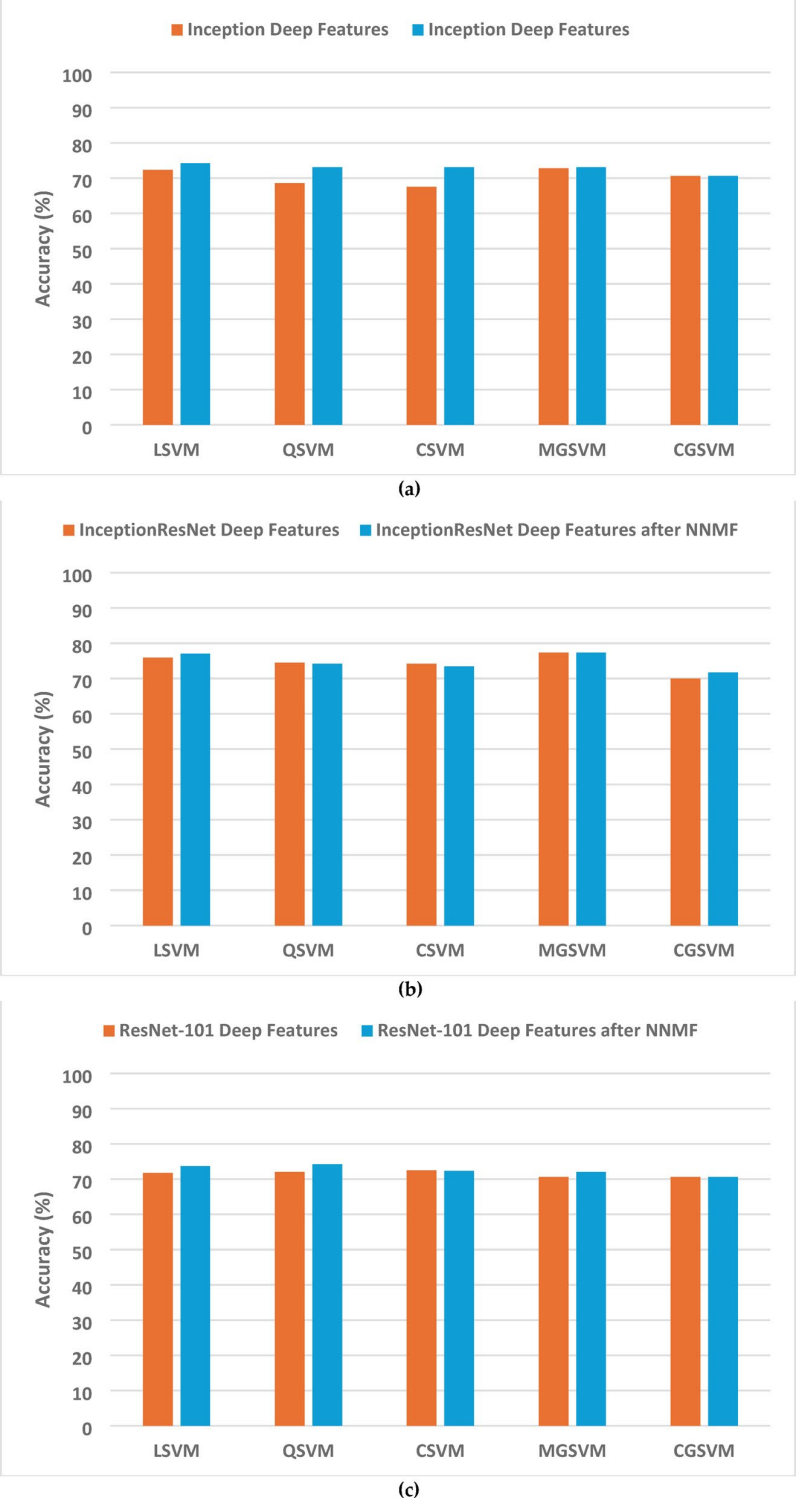
Classification accuracy comparison before and after applying the NNMF method for the three CNNs trained on the second dataset (benign versus malignant detection task): (**a**) Inception, (**b**) ResNet-101, and (**c**) InceptionResNet.

Figure 8 depicts the effect of implementing NNMF on the detection accuracy of the three CNN models trained on the second dataset for the benign versus malignant classification task. The incorporation of NNMF into the Thermo-CAD framework resulted in significant enhancements in classification efficacy across the majority of SVM classifiers. The findings illustrated in both Fig. 8; Table 8 demonstrate that NNMF-based feature transformation either preserved or improved accuracy in most instances. For instance, Inception demonstrated enhanced accuracy throughout all SVM classifiers subsequent to NNMF, while ResNet-101 consistently attained robust or improved performance following dimensionality reduction. Despite minor declines in accuracy for certain classifiers, InceptionResNet’s performance remained largely unaffected, with these alterations being negligible in terms of the model’s overall efficacy.

Significantly, NNMF facilitated a substantial decrease in feature dimensionality, converting the original highly dimensional vectors (2048 features for Inception and ResNet-101, and 1536 for InceptionResNet) to as few as 10 to 50 features. This compression markedly reduced computational requirements and mitigated the risk of overfitting, while maintaining or enhancing classification performance. NNMF demonstrates itself as a potent and efficient feature transformation method within the Thermo-CAD framework, providing an optimal equilibrium between diagnostic precision and computational efficiency, especially advantageous in resource-constrained environments.

Table 9 presents the most promising results, illustrating the performance subsequent to the use of the feature selection method on the aggregated deep features from all three networks. The classification accuracy demonstrated a consistent increase with the increase of selected features, attaining a maximum of 79.3% with CSVM with 160 features. Additional significant outcomes comprise 75.6% and 75.9% achieved with both LSVM and QSVM employing 90 and 120 features and 77.9% attained with MGSVM employing 120 features.

**Table 9:** Second dataset (benign versus malignant detection task) SVMs classification accuracy (%) accomplished with the chosen attributes subsequent to the application of Relief-F feature selection on the aggregated deep features from the three networks for the second dataset (benign versus malignant).

Model	LSVM	QSVM	CSVM	MGSVM	CGSVM
10	74.5	70.6	71.1	73.7	73.4
20	74.8	71.7	70.9	73.7	73.7
30	75.1	73.4	72.3	73.9	72.5
40	74.5	73.7	75.6	74.8	72.8
50	74.2	75.1	76.5	74.2	72.5
60	72.5	74.5	75.1	74.2	71.4
70	73.4	74.2	73.4	74.8	72.3
80	74.5	72.5	73.9	75.4	72.5
90	75.6	73.9	77.3	75.9	71.7
100	74.8	74.5	75.9	77.0	72.0
110	74.5	75.1	76.2	77.6	71.4
120	75.1	75.9	76.5	77.9	71.4
130	74.5	75.1	77.0	76.2	71.4
140	74.2	74.8	78.4	77.6	71.4
150	74.2	75.4	78.4	76.8	71.4
160	74.2	74.5	79.3	77.9	71.1
170	75.1	75.6	78.2	75.4	70.9
180	75.1	73.9	77.6	74.8	70.9
190	74.8	75.6	75.9	75.1	70.9

Further evaluation metrics are calculated for the greatest accuracy attained in Table 9 for benign and malignant detection tasks and presented in Table 10. Furthermore, the confusion matrices for the CSVM, which attained the highest performance, are presented in Fig. 6. Additionally, the ROC curve for the corresponding classifier is illustrated in Fig. 7. The AUC is also calculated and presented in Fig. 7. The findings in the benign against malignant breast cancer detection task emphasise the efficacy and limitations of the Thermo-CAD model in distinguishing between these two classifications. Table 10 presents the evaluation scores for five SVM classifiers trained on features selected via the Relief-F feature selection technique, in conjunction with deep features derived from three CNN architectures. The CSVM exhibited the highest performance among classifiers, attaining a sensitivity of 0.8968, specificity of 0.5429, precision of 0.8248, F1-score of 0.8593, and MCC of 0.4743. The data demonstrate that CSVM was the most capable classifier in detecting malignant cases, rendering it a promising option for this classification task.

Although the CSVM demonstrates relatively acceptable performance, the overall findings indicate that distinguishing malignant from benign cases is more difficult than differentiating normal from abnormal cases. Sensitivity scores are elevated across the majority of classifiers, with MGSVM attaining the highest score of 0.9444 and CGSVM reaching 0.9762, signifying that the classifiers are adept at detecting malignant cases. Nonetheless, specificity scores are inadequate, with CSVM attaining a specificity of 0.5429, while other classifiers, including CGSVM, exhibit scores as low as 0.1619. The diminished specificity values indicate that the classifiers struggle to reliably recognise benign cases, potentially resulting in an increased incidence of false positives in practice.

**Fig. 9:**
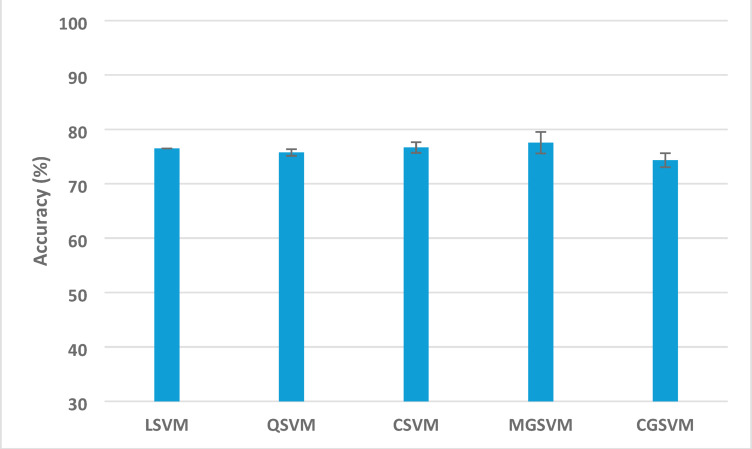
Classification accuracy with confidence intervals (CI) for the SVM classifiers trained with the selected features which achieved the highest performance after applying the Releif-F feature selection method for the second dataset.

Figure 9 illustrates the classification accuracy along with the associated confidence intervals (CI) for the five SVM classifiers built on the most discriminating attributes identified by the Relief-F method in the second dataset. Across the classifiers, MGSVM attained the highest mean accuracy of 77.54%, accompanied by a relatively narrow confidence interval (76.25–78.83%), signifying robust performance and consistency. CSVM achieved a mean accuracy of 76.66%, but its broader confidence interval (74.69–78.63%) indicates greater variability. LSVM and QSVM exhibited medium mean accuracies of 76.48% and 75.74%, respectively, accompanied by narrow and stable confidence intervals, whereas CGSVM demonstrated the lowest mean accuracy of 74.34% with the most constricted confidence interval (73.87–74.81%), indicating consistent yet inferior predictive performance across trials.

**Table 10:** Second dataset (benign versus malignant detection task) evaluation measures computed for the SVM classifiers developed with the chosen attributes following the application of Relief-F feature selection on the aggregated deep features from the three networks.

Metric	LSVM	QSVM	CSVM	MGSVM		CGSVM
Sensitivity	0.9087	0.8889	0.8968	0.9444		0.9762
Specificity	0.3905	0.4476	0.5429	0.3810		0.1619
Precision	0.7816	0.7943	0.8248	0.7855		0.7365
F1-score	0.8404	0.8390	0.8593	0.8577		0.8396
MCC	0.3554	0.3764	0.4743	0.4138		0.2563

**Fig. 10:**
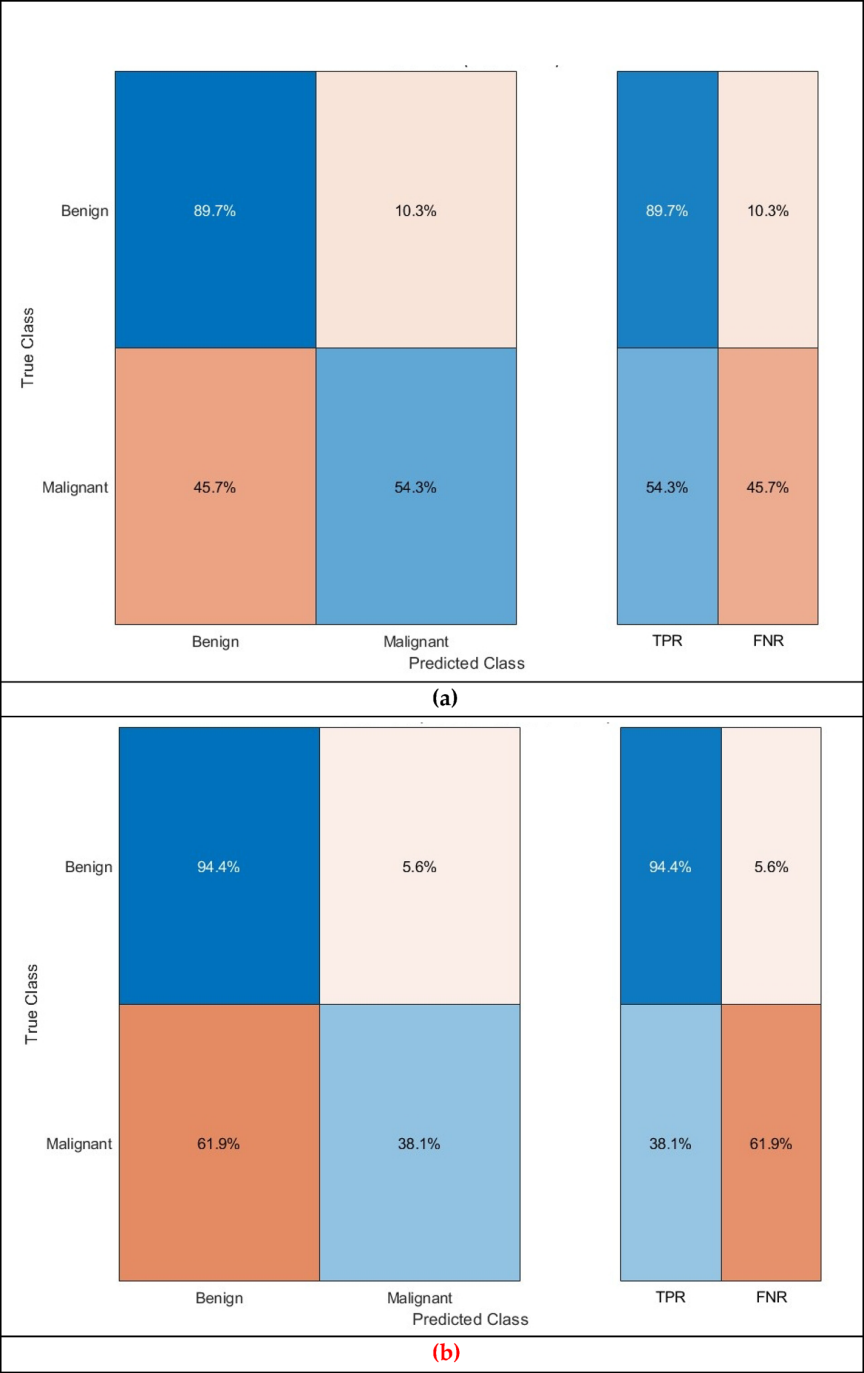

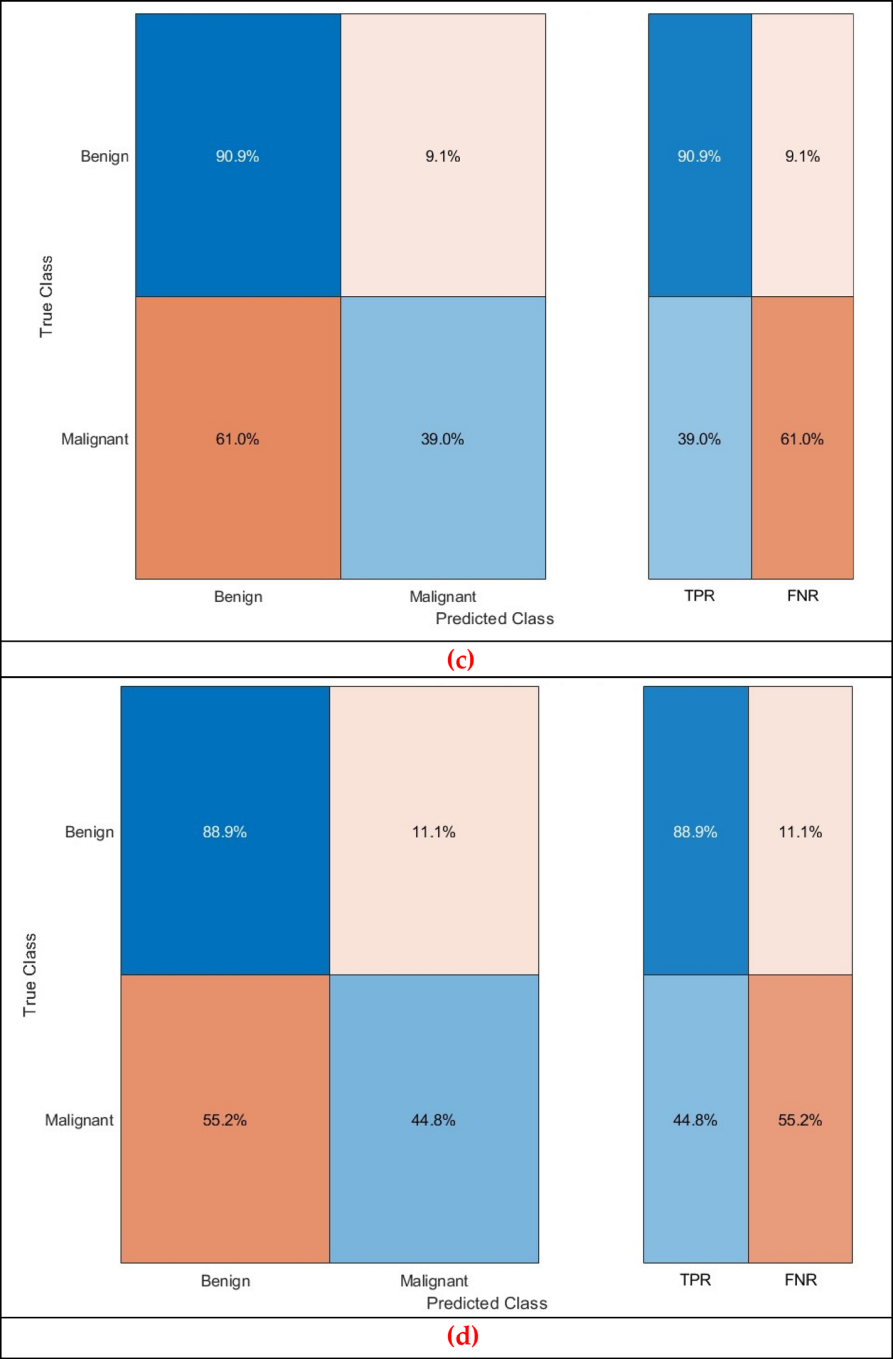
Second Dataset (benign versus malignant detection task confusion matrix for the (**a**) CSVM, (**b**) MGSVM. (**c**) LSVM, and (**d**) QSVM classifiers after the Relief-F feature selection step.

Figure 10 displays the confusion matrix for CSVM, MGSVM, LSVM, and QSVM, which visually demonstrates the likelihood of false positives. The classifier correctly identifies malignant cases but erroneously categorises a considerable number of benign cases as malignant. This finding indicates that the Thermo-CAD system, although proficient in detecting malignancies, may necessitate additional alterations in differentiating benign cases.

**Fig. 11:**
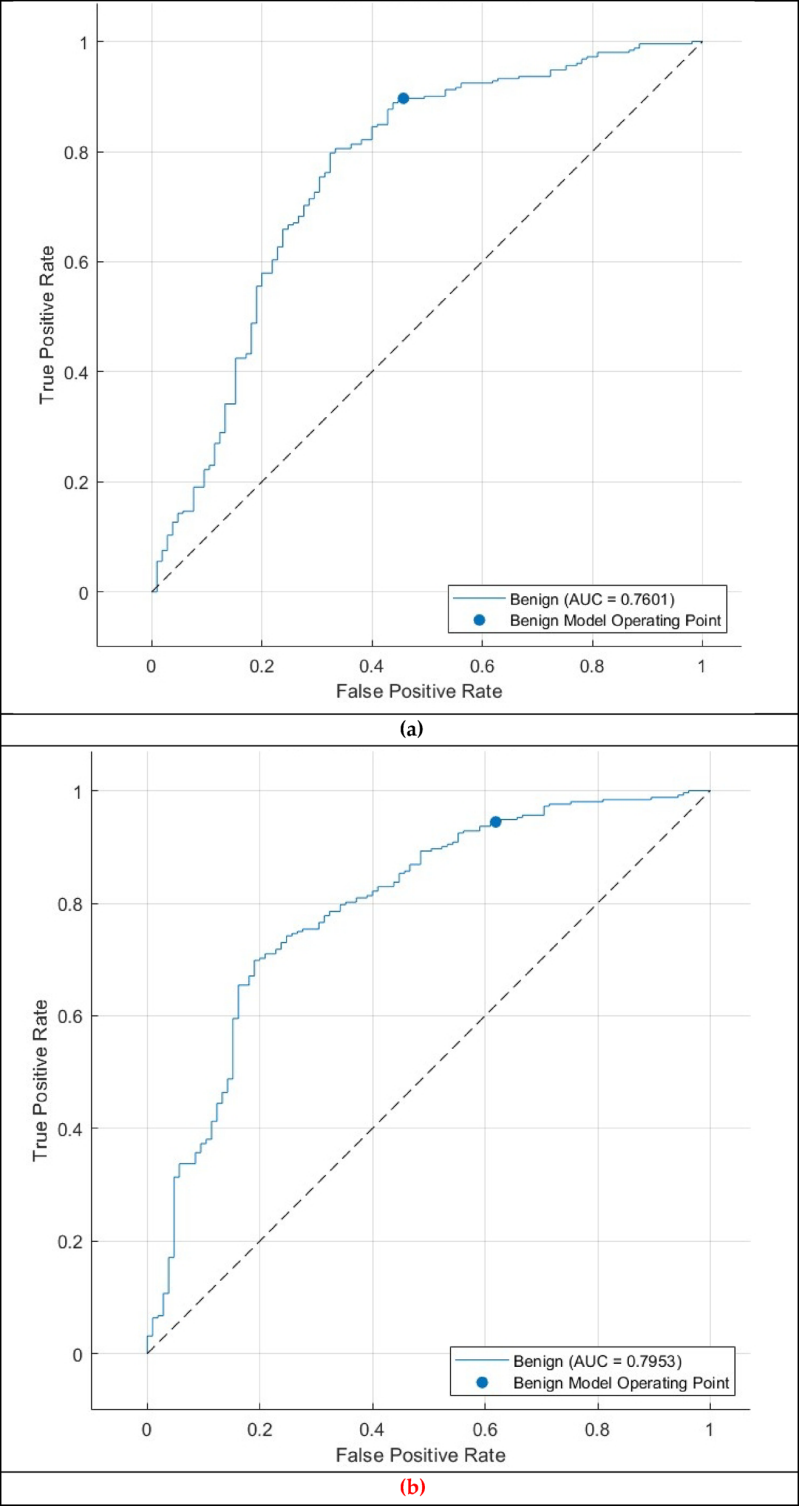

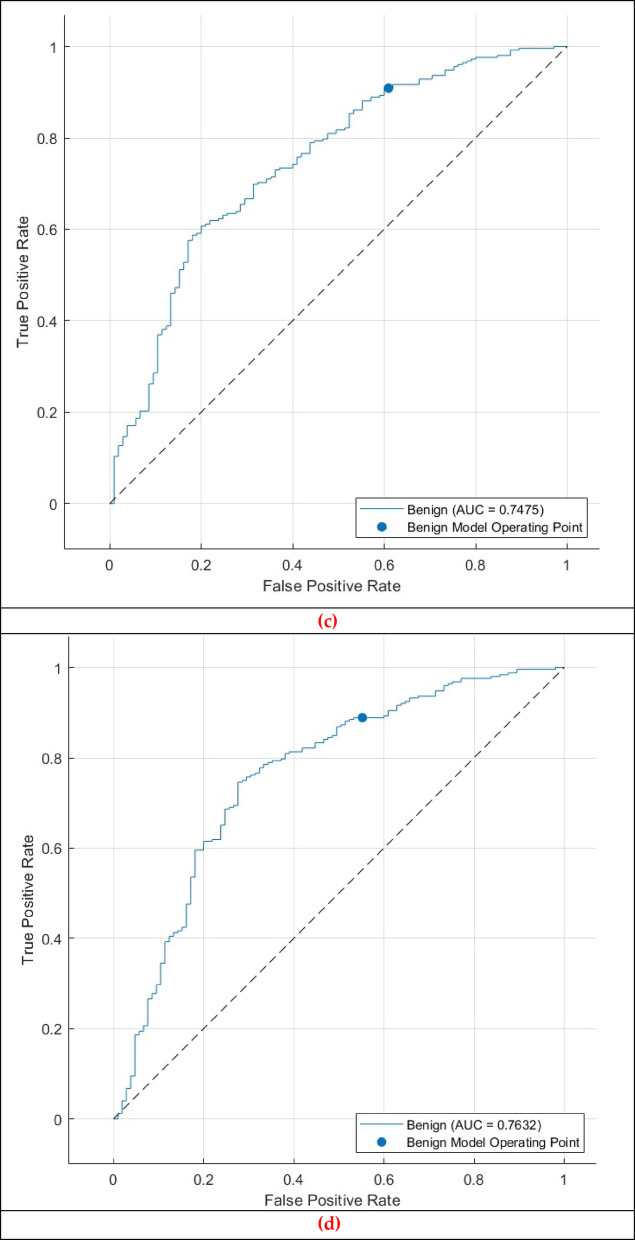
Second Dataset (benign versus malignant detection task ROC curve for the (**a**) CSVM, (**b**) MGSVM. (**c**) LSVM, and (**d**) QSVM classifiers after the Relief-F feature selection step.

The ROC curve for the CSVM, MGSVM, LSVM, and QSVM classifiers, illustrated in Fig. 11, reinforces the classifier’s dependability in distinguishing between benign and malignant cases. The AUC of 0.7601 reflects the CSVM’s overall capacity to balance sensitivity and specificity at various decision thresholds. The ROC curve and AUC indicate that the model possesses moderate discriminatory ability despite the difficulties in distinguishing between benign and malignant cases.

Contrary to the outstanding outcomes achieved in the normal-abnormal classification task, the Thermo-CAD framework demonstrated diminished proficiency in differentiating between benign and malignant tumours, as indicated by the results shown in Tables 7, 8, 9 and 10; Figs. 8, 9, 10 and 11.

## Discussion

The Thermo-CAD framework signifies a notable improvement in breast cancer detection through thermographic imaging, overcoming prevalent challenges in preprocessing and segmentation to optimise and elevate diagnostic precision. Thermo-CAD avoids laborious preprocessing and segmentation by leveraging transfer learning and sophisticated CNN architectures—specifically, ResNet-101, Inception, and InceptionResNet—for straightforward feature extraction. This method reduces the computational effort carried out in the pre-processing and segmentation stages and improves diagnostic reliability. Thermo-CAD incorporates NNMF for feature transformation and reduction, enhancing comprehension and diminishing dimensionality. Additionally, the Relief-F feature selection technique enhances the feature set by preserving just the most discriminatory features, thus optimising performance.

In the primary detection task of differentiating normal from abnormal breast tissue, Thermo-CAD exhibited exceptional performance, attaining 100% performance throughout all evaluation metrics: accuracy, sensitivity, specificity, precision, and F1-score using the selected features of the combined deep features of the three CNNs. This optimal outcome highlights Thermo-CAD’s dependability and efficacy in early detection, where the accurate identification of abnormalities is essential. The model’s complete sensitivity guarantees that no abnormal occurrences remain undetected, fulfilling a vital requirement in early-stage assessment by minimising the possibility of false negatives and facilitating prompt intervention. Similarly, 100% specificity ensures that all normal cases are accurately recognised, eliminating false positives that may result in superfluous follow-up procedures and patient distress. The outstanding precision and F1-score metrics demonstrate the model’s equilibrium between accuracy and sensitivity, confirming that each identified abnormal occurrence is genuinely positive. This degree of reliability enables Thermo-CAD to function as an efficient screening technique that alleviates clinician burden by diminishing the necessity for re-evaluation and superfluous testing, thereby concentrating resources on confirmed abnormal cases. Moreover, this consistent accuracy bolsters patient confidence in thermography as a reliable screening technique, facilitating its implementation in clinical environments and promoting early detection initiatives.

The second detection task, which entails distinguishing benign from malignant breast cancer, poses an important challenge owing to the complex variations among the two categories in thermographic photos. Thermo-CAD’s performance indicated that integrating CNN-derived features from various topologies with Relief-F feature selection markedly enhances diagnostic efficacy. The optimal configuration attained 79.3% accuracy utilising CSVM with Relief-F-selected features, highlighting Thermo-CAD’s proficiency in identifying essential discriminating attributes required for differentiating malignancies. The improved accuracy indicates that merging various feature representations from distinct CNN structures, including Inception, InceptionResNet, and ResNet-101, preserves distinctive data characteristics that may remain obscured in a singular CNN methodology.

Notwithstanding this accomplishment, Thermo-CAD’s efficacy in distinguishing benign from malignant tumours was inferior to its performance in differentiating normal from abnormal cases, underlining its relatively diminished capacity for discrimination among benign and malignant occurrences. The diminished efficacy in the benign-malignant differentiation task, relative to the normal-abnormal classification, can be ascribed to the fundamental closeness in thermal patterns between benign and malignant breast tumours. Benign and malignant lesions may display similar thermal signatures, as both can be linked to heightened blood flow, angiogenesis, and metabolic activity in the impacted areas. The significant similarity in thermal properties complicates the Thermo-CAD framework’s ability to accurately differentiate between these two cancer types using only thermal imaging data. The classification task of benign versus malignant may necessitate a more sophisticated and extensive array of features than those adequately represented by the individual CNN architectures or the feature transformation and selection methods utilised in this study. This is due to the sensitivity to feature dimensionality indicated in Tables 8, 9 and 10 implies that the thermal patterns distinguishing benign from malignant tumours are intricate and may require meticulous curation of feature sets to enhance diagnostic accuracy. Although the Thermo-CAD framework demonstrates potential in detecting malignant cases, the findings indicate that differentiating benign cases continues to be problematic due to reduced specificity scores. The CSVM classifier exhibits the optimal balance of metrics; however, additional optimisation is required to minimise false positives. These findings point out the potential of Thermo-CAD as a diagnostic tool, while also highlighting areas for enhancement to attain more equitable performance in clinical applications.

The 100% accuracy achieved using the DMR-IR dataset pertains exclusively to the efficacy of the suggested Thermo-CAD framework on the DMR-IR dataset in the binary classification task of distinguishing between normal and abnormal breast thermal pictures. This performance was attained under controlled experimental conditions utilizing a well-organized and preprocessed publicly accessible dataset featuring distinct class boundaries. This 100% accuracy was attained solely after the implementation of a comprehensive multi-stage procedure of deep feature extraction from various pre-trained CNNs, succeeded by non-negative matrix factorization (NNMF) for feature transformation and dimensionality reduction, and ultimately Relief-F for the selection of the most discriminative features. However, the accuracy attained before this multi-stage procedure was lower than 100%. On the other hand, after applying the comprehensive multi-stage procedure, with this fine-tuned feature set, just two classifiers—cubic SVM (CSVM) and medium Gaussian SVM (MGSVM)—achieved 100% accuracy; other classifiers performed lower under the same settings and feature set. The results indicate that the reported accuracy is not a global finding but rather reliant upon a thoroughly defined combination of feature extraction, feature selection, classifiers, and data characteristics.

Furthermore, the 100% accuracy should be interpreted strictly within the limited context of the DMR-IR dataset, which predominantly contains images exhibiting distinct thermal anomalies, enabling the model to acquire highly discernible patterns with great precision. The architecture of Thermo-CAD was specifically crafted to improve discriminative learning by incorporating various CNN representations and reducing redundancy via feature selection. However, the study does not assert that the model is universally applicable or that the issue of breast cancer detection via thermography has been resolved. Conversely, we recognize in the manuscript that the observed performance is likely enhanced by the relative homogeneity of the DMR-IR dataset. To evaluate the generalizability of Thermo-CAD, a secondary assessment was performed on a recently released thermographic dataset for a more complex clinical task: distinguishing between benign and malignant breast tumors. The model attained a more realistic accuracy of 79.3%, emphasizing the difficulties of this classification challenge and the constraints of relying solely on thermal imaging. These outcomes emphasize the necessity for further validation and model enhancement, especially for tasks involving complex or overlapping thermal characteristics.

### Explainability analysis

**Fig. 12:**
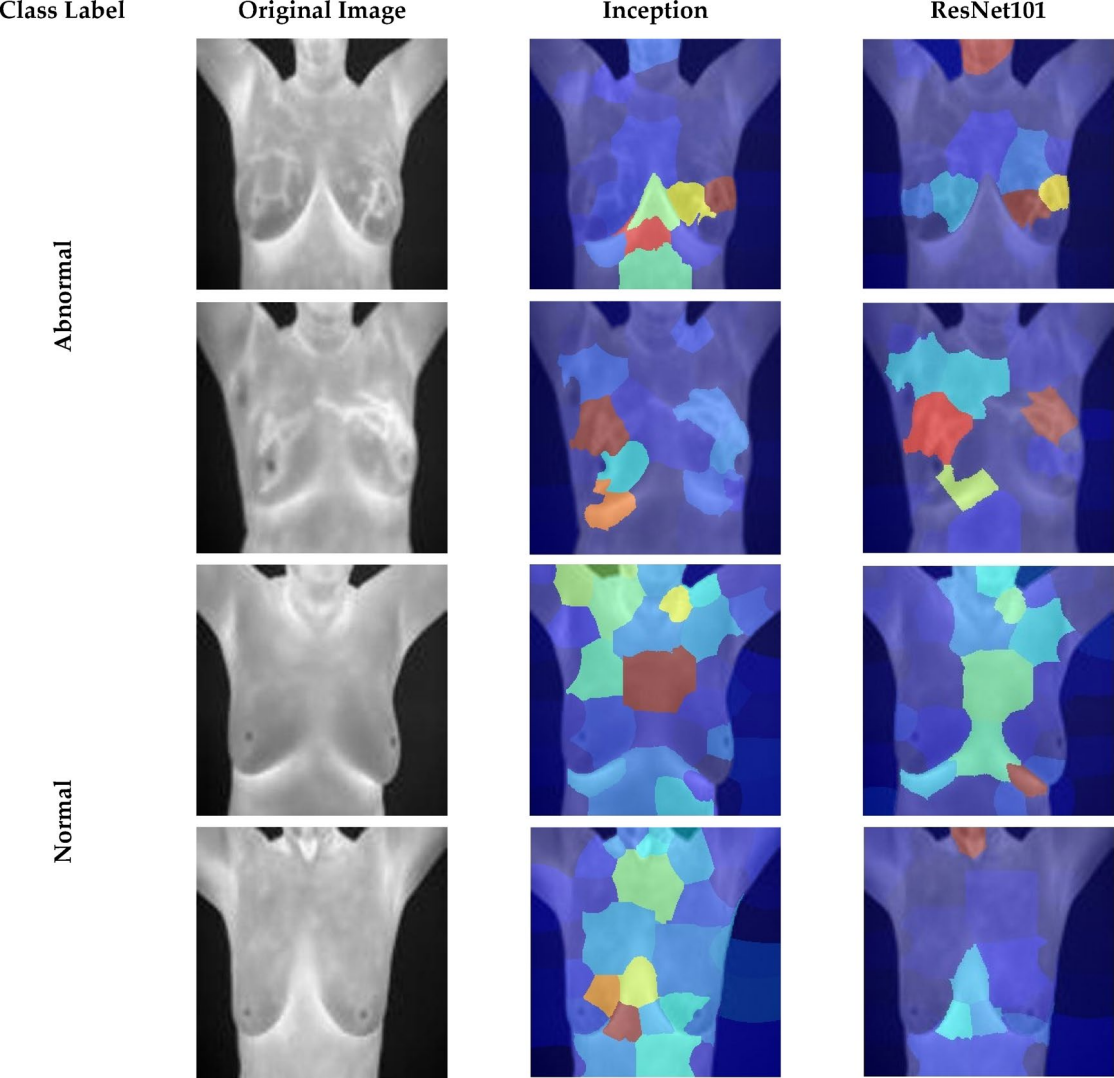
LIME heatmaps clarifying how CNNs of Thermo-CAD makes decisions regarding the class label (abnormal versus normal) of the first dataset.

This study employs the LIME (Local Interpretable Model-agnostic Explanations) method to improve the interpretability of CNN models utilized in Thermo-CAD and to elucidate how decisions are made. LIME is employed to illustrate the most significant thermal areas impacting classification results, thereby enhancing transparency and fostering clinical confidence in the model’s predictions. The heatmaps produced by LIME superimpose regions with significant relevancy onto the original thermal imaging, illustrating where the models concentrated their attention in differentiating across normal and abnormal breast tissue. Figure 12 illustrates visual clarifications produced by the LIME method for the first dataset which contains normal and abnormal images. When the LIME heatmaps are examined, it becomes clear that each CNN architecture reliably found thermal patterns linked to abnormal occurrences that were clinically significant. In the abnormal class instances, the models identified areas demonstrating localized temperature elevations, especially around the nipple and adjacent mammary areas, generally linked with angiogenesis and heightened metabolic activity related to tumor progression. These outcomes correspond with clinical knowledge, as abnormal thermograms frequently exhibit asymmetrical heat distribution or hotspots that signify underlying pathological alterations. In contrast, the normal class displayed heatmaps with more uniform and symmetrical thermal distributions throughout both breasts, featuring minor focal hotspots, which aligns with the anticipated thermal characteristics of healthy breast tissue. The symmetry and absence of significant thermal variation were crucial attributes utilized by the CNNs in recognizing normal cases.

The uniformity of attention regions among various CNN models further substantiates the resilience of the Thermo-CAD framework. Notwithstanding architectural variations among CNNs identified analogous anatomical regions as essential for classification, indicating that the acquired features are both discriminative and biologically plausible. This association strengthens the model’s reliability, as it indicates that the deep learning elements are dependent on clinically relevant thermal indicators rather than artifacts or extraneous image features. Moreover, LIME-based visualizations offer a crucial transparency for medical AI systems, allowing clinicians to comprehend and verify the rationale behind each prediction.

**Fig. 13:**
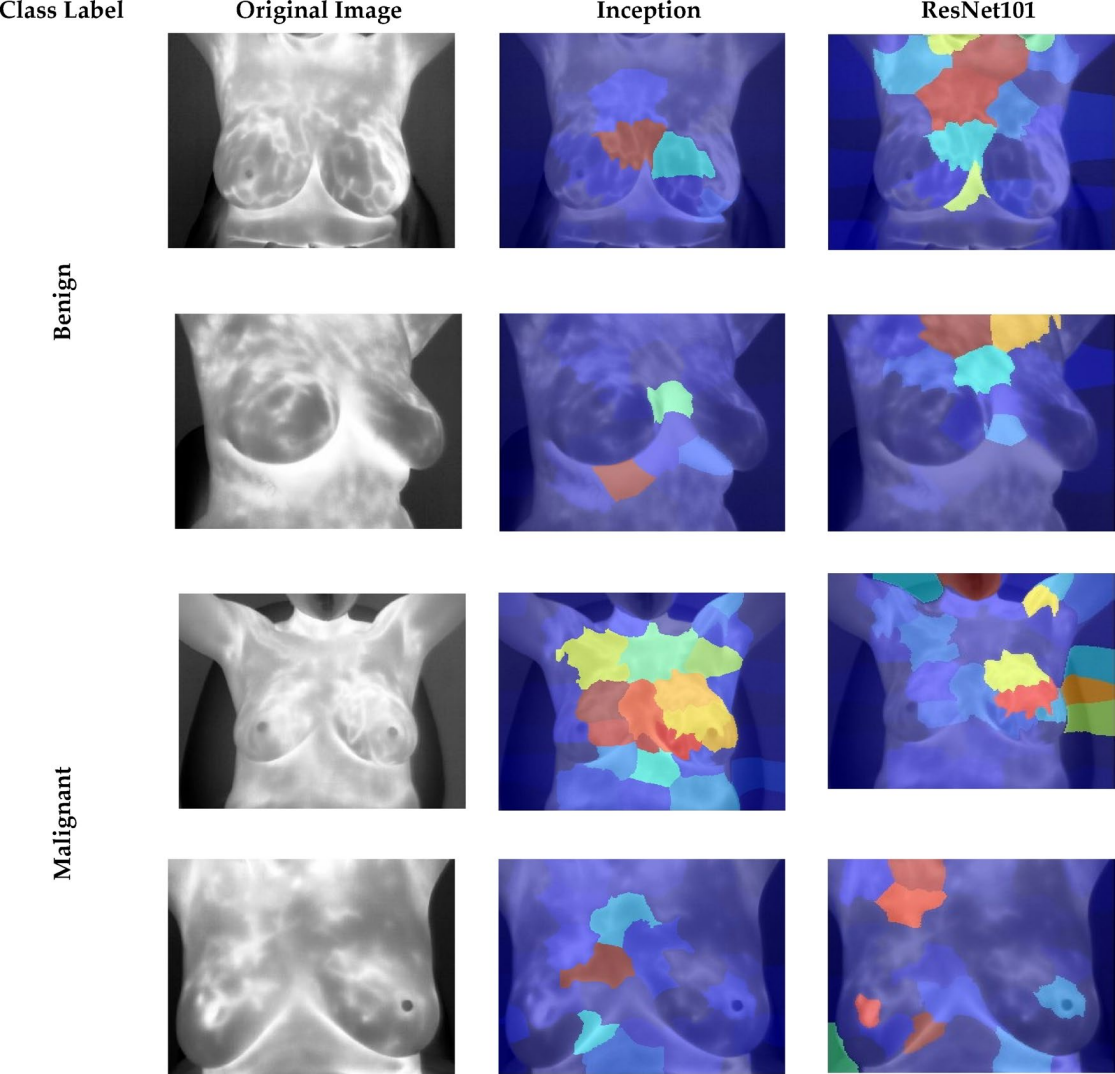
LIME heatmaps clarifying how CNNs of Thermo-CAD makes decisions regarding the class label (Malignant vs. Benign) of the second dataset.

Figure 13 offers vital insights into the decision-making procedure of the CNN models within the Thermo-CAD framework for differentiating between benign and malignant breast thermograms in the second dataset. The LIME heatmaps illustrate the areas of significance that each CNN structure emphasized during classification.

Conversely, in benign cases, the heatmaps produced by CNNs constantly emphasise symmetrical and non-aggressive thermal structures, which correspond with the common features of non-cancerous cases, including limited vascular activity and symmetrical thermal gradients. The alignment elucidates the model’s elevated sensitivity (89.68%), as it proficiently differentiates benign cases with little inconsistency. In malignant cases, CNNs frequently misclassify irregular vascular patterns or asymmetric thermal hotspots—characteristics of malignancy—as benign inflammation or normal metabolic variations. The overlap results in misclassification and reduced specificity, indicating the model’s constrained capacity to accurately detect malignant cases, which remains an important obstacle in thermographic examination.

### State-of-the-art comparisons

This section shows the results of Thermo-CAD compared to previous works for breast cancer detection using thermogram images. It is worth noting that the first dataset, entitled DMR-IR dataset for abnormal versus abnormal detection task is a benchmark dataset that has been widely used. While for the benign versus malignant detection task, the second dataset used for this purpose is a new dataset that has been recently published. To the best of the author’s knowledge, this is the first study to use this dataset to train deep learning models and investigate the models’ ability to distinguish between benign and malignant breast cancer based on thermograms. Therefore, there was no previous work to compare with in this case. Table 11 illustrates the efficacy of Thermo-CAD in breast cancer detection through thermographic images and offers a comparative assessment with prior research. In the classification task of normal versus abnormal on the DMR-IR dataset, Thermo-CAD exhibits outstanding performance, attaining 100% in all critical metrics: accuracy, sensitivity, specificity, precision, and F1-score. This performance level highlights Thermo-CAD’s capability to detect abnormal thermal patterns linked to potential malignancies without misclassifications. Conversely, alternative methods, despite attaining high accuracy, failed to achieve a perfect score across all metrics. Prior research^30^ using VGG16 in conjunction with a sequential classifier demonstrated an accuracy of 99.4% and a sensitivity of 100%, yet with a marginally reduced specificity of 97.5%. A technique employing ResNet34^57^ alongside chi-square feature selection and SVM attained a high accuracy of 99.62%, yet it did not reach the outstanding results exhibited by Thermo-CAD across all metrics. A custom CNN-based method integrated with the curvature function^41^ attained 100% accuracy and sensitivity; however, Thermo-CAD’s extensive feature selection and transformation utilising NNMF resulted in its enhanced, balanced performance across all evaluation metrics.

Thermo-CAD’s 100% performance in terms of sensitivity and specificity suggests a very dependable detection method that does not produce any false positives or negatives in the normal versus abnormal detection task. This result is crucial for clinical applications, as it decreases the likelihood of overlooked diagnoses while simultaneously minimising wasteful follow-up testing. The efficacy of Thermo-CAD is due to the combination of characteristics from various CNN architectures (Inception, ResNet-101, and InceptionResNet), the utilisation of the NNMF feature transformation and Relief-F feature selection, which proficiently identifies discriminative thermal features pertinent to breast cancer detection.

In the second classification task of differentiating benign from malignant cases, Thermo-CAD as mentioned before utilised a newly published dataset that had not been previously examined, thereby becoming the first to assess deep learning models for this objective. In this dataset, Thermo-CAD attained an accuracy of 79.3%, a sensitivity of 89.7%, and a specificity of 54.3%. The high sensitivity demonstrates a robust capacity to identify malignant cases, while the reduced specificity indicates difficulties in accurately distinguishing benign cases. The specificity of 54.3% indicates a propensity for false positives, wherein benign cases are erroneously classified as malignant, potentially leading to unnecessary follow-ups. Given this drawback, the elevated sensitivity is advantageous, as it increases the likelihood of detecting malignant cases.

**Table 11:** State-of-the-art comparisons of previous works versus Thermo-CAD.

Study	Dataset/ Task	Segmentation	Feature Selection	Methods		Accuracy	Sensitivity	Specificity	Precision	F1-score
^34^	DMR-IR Normal vs. abnormal	U-Net	No	Customised CNN		0.9933	1.00	0.9867		
^35^	DMR-IR Normal vs. abnormal	Fuzzy C-means Clustering	No	Customised EDCNN		0.9680		0.9370		
^32^	DMR-IR Normal vs. abnormal	Temperature Ranges	No	Customised CNN		0.9385	0.9053	0.9700	0.9666	
^30^	DMR-IR Normal vs. abnormal	No	No	VGG16 + Sequential Classifier		0.9940	1.00	0.9750	0.9890	0.9980
^41^	DMR-IR Normal vs. abnormal	curvature function *k* and the gradient vector flow		Customized CNN		1.00	1.00	1.00	1.00	1.00
^42^	DMR-IR Normal vs. abnormal	No	No	Customised CNN		0.970	1.00	0.830		
^58^	DMR-IR Normal vs. abnormal	level-set segmentation	No	GLCM + GLRM + GLSZM + NGTDM + PCA + SVM		0.960	1.00	0.920		
^43^	DMR-IR Normal vs. abnormal	Canny Edge Detector + Morphological Operations +	No	AlexNet		0.9048	0.9333	0.8333	0.9333	
^38^	DMR-IR Normal vs. abnormal	Morphological operation + objected oriented segmentation	No	Customised CNN		0.9895	0.9828	0.9959	0.9956	
^57^	DMR-IR Normal vs. abnormal	No	Yes	ResNet34 + Chi-square + SVM		0.9962	0.9963		0.9963	0.9963
Thermo-CAD	DMR-IR Normal vs. abnormal	No	Yes	Inception + ResNet101 + InceptionResNet + NNMF + Releif-F + SVM	80 features	1.000	1.000	1.000	1.000	1.000
	Second Dataset: Benign vs. malignant			Inception + ResNet101 + InceptionResNet + NNMF + Releif-F + SVM	160 features	0.7930	0.8969	0.5429	0.8248	0.8593

### Limitations and future directions

The Thermo-CAD system exhibits potential in breast cancer detection; however, various limitations are identified that could affect its practical application and efficacy. A notable limitation is the model’s diminished specificity in differentiating benign from malignant cases. The system demonstrates high sensitivity, reflecting its robust capacity to detect malignant occurrences; however, the reduced specificity results in a heightened incidence of false positives, wherein benign tumours are incorrectly classified as malignant, which poses a risk of false positives. These erroneous alerts may lead clinicians to advocate for invasive interventions, such as biopsies, which may be unnecessary. From a clinical perspective, reducing such incidents is crucial to guarantee that patients receive precise, effective, and comforting care. This limitation indicates that the Thermo-CAD framework, in its present form, may necessitate additional refinement to successfully maintain sensitivity and specificity. These enhancements are crucial for minimising superfluous follow-up processes in clinical environments, which may otherwise elevate anxiety among patients and healthcare expenditures. To alleviate these risks, one promising solution is the incorporation of clinician-in-the-loop systems within the Thermo-CAD framework. In this configuration, Thermo-CAD would serve as a decision support test rather than an independent diagnostic system. The model’s output, especially in ambiguous or low-confidence scenarios, will be evaluated and interpreted by a radiologist or clinician prior to any clinical intervention. This collaborative method guarantees that AI-generated predictions are informed by expert assessment, minimizing the risk of superfluous procedures while preserving the advantages of early detection. Furthermore, the incorporation of multi-modal imaging, such as the amalgamation of thermographic data with mammography or ultrasound, can supply the model with enhanced contextual information, thus boosting the ability to distinguish between benign and malignant cases.

Furthermore, the study did not employ any class-imbalance mitigation problem method like Synthetic Minority Over-sampling Technique (SMOTE) or class-weighted loss functions during the training phase of the deep learning models. The lack of a class imbalance mitigation approach may have affected classification performance. Future endeavors will incorporate techniques such as SMOTE, adaptive resampling methods, or class-weighted loss functions. Integrating these methodologies is anticipated to improve the model’s equilibrium between sensitivity and specificity, especially in datasets with imbalanced class distributions, thus enhancing its clinical relevance and dependability in differentiating between benign and malignant breast tumors through thermal imaging. As mentioned before, a significant false positive rate may result in unneeded biopsies and further diagnostic interventions, thereby elevating healthcare expenses and increasing patient anxiety and emotional turmoil. This issue highlights the necessity of improving the practical implementation of the Thermo-CAD system in actual clinical environments. Therefore, Thermo-CAD is considered not as an independent diagnostic approach, but as a supplementary tool that can be incorporated with current diagnostic modalities like mammography and ultrasound. Thermography, utilized in Thermo-CAD, provides the benefits of being non-invasive, devoid of radiation, and economically viable, rendering it especially attractive for preliminary screening or in resource-constrained settings. Nonetheless, its restricted specificity, particularly in differentiating benign from malignant abnormalities, underscores the necessity for a multimodal diagnostic strategy.

In subsequent research, the study will consider the integration of a multi-modal fusion strategy that integrates thermographic data with structural imaging data from modalities such as mammography or additional clinical variables. By utilizing the distinct advantages of each imaging technique, such as the superior spatial resolution of mammography and the metabolic and vascular information from thermography, a more thorough and precise evaluation of breast cancer can be delivered. This integrative methodology would enable Thermo-CAD to function as an early warning system or screening method in which cases identified as suspicious through thermography could undergo further assessment via mammography or ultrasound prior to the recommendation of any invasive procedures.

Furthermore, employing ensemble learning or hybrid architectures that integrate image-level and feature-level data from various sources may augment the model’s capacity to differentiate subtle variations between benign and malignant lesions, thus enhancing specificity. This multi-modal approach offers a promising avenue to diminish false positives, alleviate unnecessary biopsies, and enhance clinician confidence in CAD-assisted breast cancer screening systems. This viewpoint aligns with the overarching objective of developing a resilient, clinically applicable diagnostic support system that augments, rather than supplants, existing diagnostic processes. Furthermore, the DMR-IR dataset used in this study may exhibit a lack of diversity (e.g., in age, ethnicity, breast density), which could potentially exaggerate performance outcomes. The study also did not investigate the application of Vision Transformers (ViT), which can capture spatial relationships in photos and could increase the precision of tumour identification tasks.

Future research will focus on investigating multi-modal strategies that integrate thermography with alternative imaging techniques. This integration may augment the model’s diagnostic capabilities and enhance its proficiency in distinguishing between benign and malignant cases with increased specificity. Furthermore, employing more sophisticated feature selection methods or incorporating domain-specific features significant to breast cancer may enhance the model’s precision. Moreover, use ViT to examine its ability to detect breast cancer using thermograms. Besides, apply class imbalance techniques to handle the imbalance problem of the second dataset. Additionally, it is advisable to evaluate the Thermo-CAD framework on larger, more heterogeneous datasets to confirm its robustness and generalisability in wider clinical applications. Patient-wise partitioning is an established methodology designed to avert data leakage and guarantee that images from the same patient are not included in both training and testing datasets, as this could otherwise lead to an inflated assessment of model efficacy.

Nonetheless, the current study was constrained by the configuration of the utilized datasets. Neither the DMR-IR nor the second thermographic dataset includes metadata that specifies the association of images with individual patients. Consequently, patient-wise splitting could not be executed with the data at hand. The datasets consist of sets of independent thermographic images categorized as normal, benign, or malignant, devoid of patient identifiers or explicit grouping details. Therefore, the training and testing division was conducted at the image level, a limitation acknowledged by the study. Future research will focus on acquiring or creating datasets that encompass comprehensive patient-level metadata, facilitating patient-specific data partitioning. This will enable a more thorough assessment of the Thermo-CAD framework’s generalization ability and enhance the simulation of its real-world application, where patient-level variability is crucial.

The study acknowledges the necessity of external validation on entirely independent datasets to enhance robustness and minimize the risk of overfitting to the characteristics of a specific dataset. Regrettably, access to publicly available thermal breast imaging datasets is constrained, and the majority of alternatives referenced in the literature are proprietary and not accessible to the public. Notwithstanding these limitations, the Thermo-CAD framework was validated on two separate publicly available datasets—DMR-IR and a newly released thermographic dataset—differing in acquisition settings, imaging protocols, and class distributions. This dual-dataset assessment offers significant external validation; however, extensive validation on more diverse, patient-labeled datasets is crucial and will be a primary focus of forthcoming research.

## Conclusions

Thermo-CAD showed great promise as a sophisticated CAD system for thermographic image-based breast cancer detection. On the extensively employed DMR-IR dataset, Thermo-CAD achieved flawless accuracy, sensitivity, specificity, precision, and F1-score, demonstrating its reliability in differentiating normal from abnormal breast tissue. Thermo-CAD’s performance surpassed previous methods, attaining a robust array of metrics that guaranteed high confidence in early-stage cancer detection. Thermo-CAD was assessed on a novel dataset for the benign versus malignant classification task, signifying it as the primary study to use this dataset for deep learning-based detection to the best of the author’s knowledge. Although the model achieved great sensitivity (89.7%), its lowered specificity (54.3%) in this work underlined the difficulty of differentiating minor thermal changes between benign and malignant cases. Although Thermo-CAD was effective at identifying anomalies and showed great potential for clinical use, the results showed that future studies should give top priority to the integration of additional imaging modalities and the improvement of feature extraction and selection techniques to increase its discriminating efficacy in malignancy detection.Thermo-CAD was a promising approach for improving breast cancer screening and diagnostics. Although the diagnostic accuracy for distinguishing benign from malignant tumors may not yet equal that of established modalities such as mammography, the framework’s ability to attain near-perfect classification in the normal-abnormal task and outstanding performance in the benign-malignant task indicated its potential as an additional screening or triage tool, especially in environments with restricted access to advanced imaging technologies.

## Data Availability

The following link provides the DMR-IR database employed for the present research, which can be downloaded upon request: https://visual.ic.uff.br/dmi/The following link provides public access to the second database utilised throughout this research: https://data.mendeley.com/datasets/mhrt4svjxc/3.

## Declarations

### Approval for animal experiments

Not applicable, as no experiments involving live vertebrates or higher invertebrates were conducted.

### Approval for human experiments

Not applicable, as the study does not include human participants or tissue samples.
